# Antitumor Activity, Mechanisms of Action and Phytochemical Profiling of Sub-Fractions Obtained from *Ulex gallii* Planch. (Fabaceae): A Medicinal Plant from Galicia (Spain)

**DOI:** 10.3390/molecules30040972

**Published:** 2025-02-19

**Authors:** Lucía Bada, Hussain Shakeel Butt, Elías Quezada, Aitor Picos, Helle Wangensteen, Kari Tvete Inngjerdingen, José Gil-Longo, Dolores Viña

**Affiliations:** 1 Group of Pharmacology of Chronic Diseases (CD Pharma), Molecular Medicine and Chronic Diseases Research Centre (CIMUS), Universidade de Santiago de Compostela, 15782 Santiago de Compostela, Spain; lucia.bada@rai.usc.es (L.B.); aitor.picos@rai.usc.es (A.P.); 2 Department of Pharmacology, Pharmacy and Pharmaceutical Technology, Faculty of Pharmacy, Universidade de Santiago de Compostela, 15782 Santiago de Compostela, Spain; elias.quezada@usc.es (E.Q.); jose.gil.longo@usc.es (J.G.-L.); 3 Section for Pharmaceutical Chemistry, Department of Pharmacy, University of Oslo, 0316 Oslo, Norway; hsb95@hotmail.no (H.S.B.); helle.wangensteen@farmasi.uio.no (H.W.); k.t.inngjerdingen@farmasi.uio.no (K.T.I.)

**Keywords:** *U. gallii*, sub-fractions, neuroblastoma, glioblastoma, cell cycle, apoptosis

## Abstract

The plant kingdom serves as a valuable resource for cancer drug development. This study explored the antitumor activity of different sub-fractions (hexane, dichloromethane and methanol) of *U. gallii* (gorse) methanol extract in glioblastoma (U-87MG and U-373MG) and neuroblastoma (SH-SY5Y) cell lines, along with their phytochemical profiles. Cytotoxicity was evaluated using 3-(4,5-dimethylthiazol-2-yl)-2,5-diphenyltetrazolium bromide (MTT) and lactate dehydrogenase (LDH) assays, and cell cycle arrest and apoptosis were assessed through flow cytometry and by measuring reactive oxygen species (ROS) and protein expression levels. D7 and D8 dichloromethane sub-fractions significantly reduced cell viability, triggered early apoptosis in SH-SY5Y and U-87MG cells and specifically increased ROS levels in U-87MG cells. Western blot analyses showed that D7 increased p53, caspase-3, caspase-8 and γH2AX expression in SH-SY5Y and U-87MG cells, while D8 specifically elevated p53 in SH-SY5Y cells and caspase-3 in both cell lines. In U-373 cells, D7 and D8 markedly reduced cell viability, with D8 inducing necrosis. Morphological changes indicative of apoptosis were also observed in all cell lines. Bioinformatic analysis of UHPLC-MS and GC-MS data tentatively identified 20 metabolites in D7 and 15 in D8, primarily flavonoids. HPLC-DAD confirmed isoprunetin and genistein as the most abundant in D7 and D8, respectively, both isolated and identified by NMR spectroscopy. Most of the flavonoids identified have been reported as antitumor agents, suggesting that these compounds may be responsible for the observed pharmacological activity.

## 1. Introduction

Cancer is one of the leading public health concerns worldwide. In 2020, over 19 million people were diagnosed, and 10 million deaths were reported [1]. The high incidence and mortality rates highlight the urgent need to intensify efforts in developing effective treatments.

The plant kingdom serves as a valuable resource for drug development. Approximately 50% of the modern drugs derived from natural products originate from plants, and about 60% of prescribed anticancer drugs are derivatives of plant metabolites. Among the most used plant-derived compounds in cancer treatment are vinca alkaloids, taxanes and anthracyclines [2,3,4]. In the mid-twentieth century, attention shifted toward synthetic compounds due to the challenges associated with natural products, such as poor water solubility, low stability, limited bioavailability and difficulties in large-scale isolation. However, recent advancements in technology and methodology offer solutions to these limitations, justifying the renewed interest in natural products [5]. This shift is further supported by concerns regarding the systemic adverse effects of synthetic drugs, the rapid development of drug resistance and their higher cost [6].

With the renewed interest in plant metabolites, the *Ulex* genus has attracted our attention for its potential pharmacological applications. *Ulex* is a genus of flowering plants in the family Fabaceae which comprises about 20 species native to Western Europe and Northwest Africa. Among them, *U. europaeus* has demonstrated antioxidant activity due to its high polyphenol content. Additionally, it inhibits α-glucosidase and α-amylase activities, effects attributed to its liquiritigenin content [7]. Furthermore, flavonoids isolated from the *Ulex* species have exhibited antifungal activity, including 7-*O*-methylisolupalbigenin from *U. airensis* and several metabolites from *U. europaeus* such as isolupalbigenin, licoisoflavone B, onogenin and 2-methoxypterocarpin [8]. Other *Ulex* species, including *U. jussiaei* and *U. minor*, have also shown antifungal properties due to their content of maackiain, 4-methoxymaackiain, 2-methoxymaackiain, isobavachromene, isolupalbigenin and ulexone A [9]. Importantly, several of these species have also shown promise in cancer research.

Among them, *U. europaeus* stands out not only as one of the most invasive species in the world but also for its potential in cancer therapy and diagnosis [10]. A water-soluble extract from *U. europaeus* seeds has been found to inhibit the growth of various reticuloendothelial tumor cell lines through a non-cytotoxic and reversible mechanism, and some types of myelomas have shown sensitivity to extracts of the same species [11,12]. *U. europaeus* has a high content of bioactive phenolic compounds that may be responsible for its antitumor activity. The predominant classes of compounds in its leaves and flowers include isoflavones (mainly glycitin), flavones (primarily apigenin derivatives), flavonols (notably quercetin glucosides) and flavanones (derivatives of liquiritigenin) [7]. Phenolic compounds, such as phenolic acids, flavonoids, tannins, stilbenes and coumarins, have been associated with cancer chemopreventive properties by inducing apoptosis through cell cycle arrest or blocking signaling pathways [13]. *U. europaeus* also offers other applications in cancer. Ulex europaeus agglutinin I (UEA-1) is a plant lectin which binds specifically to α-l-fucose moieties on the surface glycoproteins of human endothelial cells, making it a useful histological marker of the endothelium in human tissues [14]. This property makes it a specific and sensitive tool for detecting the presence of endothelial cells in certain human tumors, such as endothelial sarcomas [15]. It is also employed as a biomarker for the human epidermal growth factor receptor 2 (HER2), a glycoprotein overexpressed in breast cancer, and as an endothelial marker in neoplastic thyroid cells [16,17]. UEA-1 has also been used to significantly discriminate between patients with benign prostatic hyperplasia and bladder cancer due to its higher binding affinity for the fucosylated-glycoisoform of integrin alpha-3 present in the urine of bladder cancer patients [18].

Additionally, other species of the *Ulex* genus have shown potential in cancer research. Extracts from the flowers and leaves of *U. parviflorus* have demonstrated cytotoxic activity against the A549 lung cancer cell line [19]. While studies have yet to establish a direct correlation between this activity and its chemical composition, 2,3,4-trimethoxy-8,9-methylenedioxypterocarpan has been identified and isolated as one of its constituents [20]. Notably, pterocarpans have been shown to induce tumor cell death through persistent mitotic arrest during the prometaphase [21].

This article focuses on the antitumor activity and phytochemical profile of *U. gallii* Planch., a medicinal plant from Galicia, Spain, for which there were no studies prior to the work of our team. Recently, we described the antiproliferative activity of polar extracts from *U. gallii* Planch. against the A549 (lung cancer) and AGS (stomach cancer) cell lines. In this study, we identified several flavonoids that may contribute to this antiproliferative effect [22].

In the present work, we extend our investigation into the antiproliferative properties of *U. gallii* to include additional tumor cell lines, aiming to elucidate its underlying mechanisms of action. We evaluated several sub-fractions of hexane, dichloromethane and methanol extracts obtained from a crude methanol extract of *U. gallii* in glioblastoma and neuroblastoma models using the U-87MG, U-373MG and SH-SY5Y cell lines. Additionally, a human normal lung fibroblast cell line, MRC-5, was incorporated in the study to assess the susceptibility of *U. gallii* sub-fractions in non-tumoral cells [23].

Malignant gliomas are the most aggressive brain tumors in adults, with a life expectancy of only 12 to 15 months [24]. Glioblastoma multiforme (GBM), a grade IV glioma, is highly invasive and treatment-resistant, with its prognosis remaining unchanged for decades, emphasizing the need for new treatments [25]. U-87MG and U-373MG cell lines are human GBM cells widely used in neuroscience and oncology research. They differ in gene expression and invasiveness [26].

Neuroblastoma is the most common extracranial tumor in children, responsible for over 15% of pediatric cancer deaths [27]. High-risk cases have limited treatment options, and current therapies often lead to the development of multidrug-resistant clones and an increased likelihood of relapse [28]. SH-SY5Y cells are human neuroblastoma cells frequently used in scientific studies as a model for neuronal activity and differentiation in vitro [29].

To elucidate the antitumoral activity of the sub-fractions from the methanol extract of *U. gallii* described herein, their phytochemical profile was determined using several strategies. Crude datasets obtained from UHPLC-MS were processed, and precursor *m*/*z* values, retention times and experimental fragmentation patterns were compared against spectral database libraries using different metabolomic platforms and bioinformatics tools. To refine the tentative identification of the sub-fractions’ composition, GC-MS analysis was employed, along with the isolation and quantification of the major compounds present in the sub-fractions.

## 2. Results

### 2.1. Several Sub-Fractions Reduced Cell Viability

**Figure 1:**
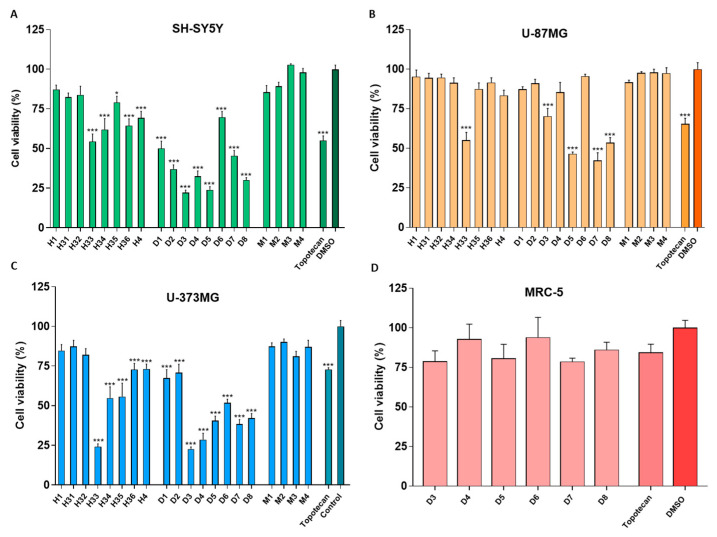
Effect of hexane (H1, H3.1–3.6, H4), dichloromethane (D1–D8) and methanol (M1–M4) sub-fractions (0.1 mg/mL) on cell viability after 24 h of treatment of the: (**A**) SH-SY5Y, (**B**) U-87MG and (**C**) U-373MG cell lines, and effect of dichloromethane (D3–D8) sub-fractions (0.1 mg/mL) on cell viability after 24 h of treatment of the (**D**) MRC-5 cell line. Topotecan (10 µM) was used as a positive control. Data represent the means ± s.e.m. (standard error of mean) from at least three independent experiments (*n* = 3). * *p* < 0.05 and *** *p* < 0.0001 compared to the respective control (cells treated with DMSO (vehicle) < 1%).

The results indicate that all active sub-fractions at 0.1 mg/mL for 24 h displayed greater toxicity towards the SH-SY5Y and U-373MG cell lines compared to the U-87MG cell line. Specifically, the hexane sub-fractions (H3.3–3.6 and H4) and dichloromethane sub-fractions (D1-D8) exhibited high toxicity against SH-SY5Y and U-373MG cells. However, only the H3.3, D3, D5, D7 and D8 subfractions demonstrated significant toxicity against the U-87MG cell line. Among all the cell lines tested, H3.3 was identified as the most potent hexane-derived sub-fraction, and D5, D7 and D8 induced higher toxicity than the positive control, topotecan, a reference antitumoral molecule (Figure 1A–C). For its part, the methanol sub-fractions (M1–M4), also at 0.1 mg/mL for 24 h, did not cause toxicity in any of the cell lines studied. Furthermore, the cytotoxic effects of sub-fractions D3-D8 were investigated on MRC5 cells, showing no significant toxic effects on non-tumoral cell viability (Figure 1D).

**Figure 2:**
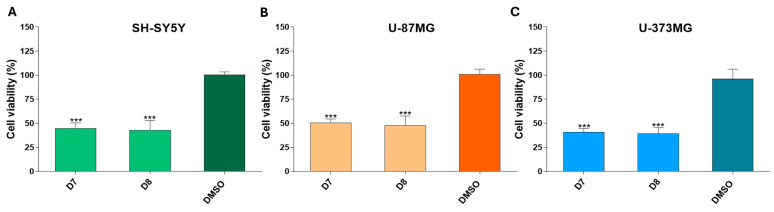
Effect of dichloromethane sub-fractions D7 and D8 (0.05 mg/mL) after 24 h of treatment on the cell viability of cell lines: (**A**) SH-SY5Y, (**B**) U-87MG and (**C**) U-373MG. Data represent the means ± s.e.m. (standard error of mean) from at least three independent experiments (*n* = 3). *** *p* < 0.0001 compared to the respective control (cells treated with DMSO (vehicle) < 0.5%).

The cytotoxicity of two of the most potent sub-fractions, D7 and D8, was also evaluated at 0.05 mg/mL for 24 h. With the new conditions, D7 and D8 inhibited cell viability by around 50% in the different cell lines: SH-SY5Y, U-87MG and U-373MG (Figure 2A–C).

### 2.2. The Different Dichloromethane Sub-Fractions (Excluding Sub-Fraction D8 in U-373MG) Did Not Compromise Membrane Integrity

**Figure 3:**
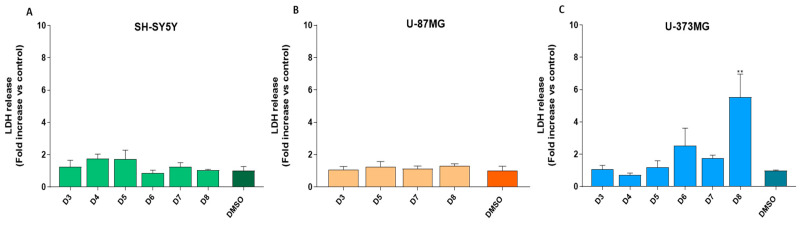
Effect of dichloromethane sub-fractions (D3–D8, 0.05 mg/mL) on LDH release after 24 h of treatment of cell lines: (**A**) SH-SY5Y, (**B**) U-87MG and (**C**) U-373MG. Data represent the means ± s.e.m. (standard error of mean) from at least three independent experiments (*n* = 3). ** *p* < 0.01 compared to the respective control (cells treated with DMSO (vehicle) < 0.5%).

LDH measurement is a widely used indicator of cell membrane integrity and cytotoxicity. After 24 h of treatment with the sub-fractions D3–D8 (0.05 mg/mL), no significant differences in LDH release were detected in SH-SY5Y and U-87MG cells compared to control cells (DMSO) (Figure 3A,B). However, sub-fraction D8 significantly increased LDH release in the U-373MG cell line (Figure 3C).

### 2.3. Sub-Fractions D7 and D8 Induced an Increase in Reactive Oxygen Species (ROS) in U-87MG Cells

**Figure 4:**
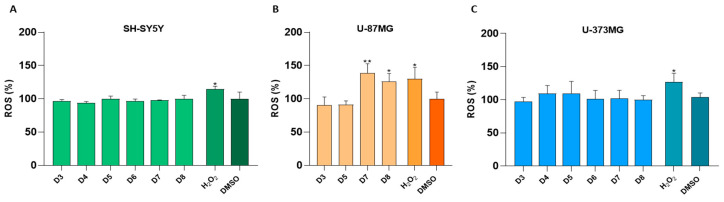
Effect of the dichloromethane sub-fractions (D3–D8, 0.05 mg/mL) on ROS production after 24 h of treatment of cell lines: (**A**) SH-SY5Y, (**B**) U-87MG and (**C**) U-373MG. H_2_O_2_ (100 µM) was used as a positive control. Data represent the means ± s.e.m. (standard error of mean) from at least three independent experiments (*n* = 3). * *p* < 0.05 and ** *p* < 0.01 compared to the respective control (cells treated with DMSO (vehicle) < 0.5%).

The effects of the dichloromethane sub-fractions D3–D8 (0.05 mg/mL) on ROS levels were evaluated in SH-SY5Y, U-87MG and U-373MG cells after 24 h of treatment. The D7 and D8 sub-fractions induced a noteworthy increase in ROS production, specifically in U-87MG cells (Figure 4A–C). Nevertheless, the control agent H_2_O_2_ significantly increased ROS levels across all cell lines.

### 2.4. Sub-Fractions D3–D8 Induced Cell Cycle Disruption

**Figure 5:**
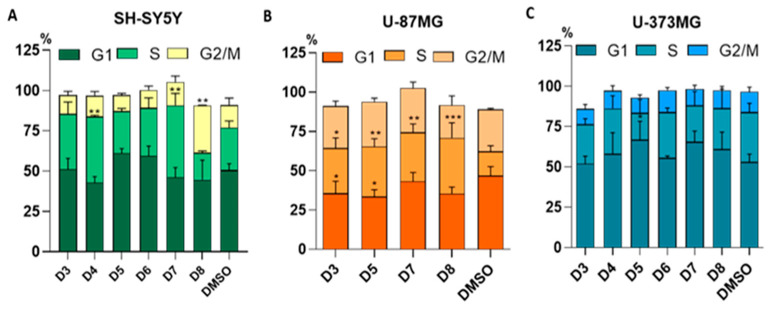
Effect of the sub-fractions D3–D8 (0.05 mg/mL) on cell cycle progression after 24 h of treatment of cell lines: (**A**) SH-SY5Y, (**B**) U-87MG and (**C**) U-373MG. Data represent the means ± s.e.m. (standard error of mean) from at least three independent experiments (*n* = 3). * *p* < 0.05,** *p* < 0.01 and *** *p* < 0.0001 compared to the respective control (cells treated with DMSO (vehicle) < 0.5%).

The cell-cycle progression was analyzed in SH-SY5Y, U-373MG and U-87MG cell lines, comparing between untreated (DMSO) and sub-fractions-treated cells (0.05 mg/mL for 24 h). In untreated SH-SY5Y and U-373MG cells, most cells were observed in the G1 and S phases, whereas untreated U-87MG cells showed a higher proportion in the G2/M phase compared to the S phase. The treatment of SH-SY5Y cells with D4 and D7 sub-fractions significantly increased the percentage of cells in the S phase, while sub-fraction D8 led to an accumulation in the G2/M phase (Figure 5A). In U-87MG cells, sub-fractions D3, D5, D7 and D8 induced a significant arrest in the S phase. Additionally, sub-fractions D3 and D5 caused a concomitant decrease in the proportion of cells in the G1 phase compared to the control (Figure 5B). However, for U-373MG, only sub-fraction D5 caused a notable increase in the proportion of cells in the G1 phase compared to the control (Figure 5C). Representative cell cycle histograms are shown in the Supplementary Material (Figure S1).

### 2.5. Sub-Fractions D7 and D8 Induced Apoptosis in SH-SY5Y and U-87MG Cells

**Figure 6:**
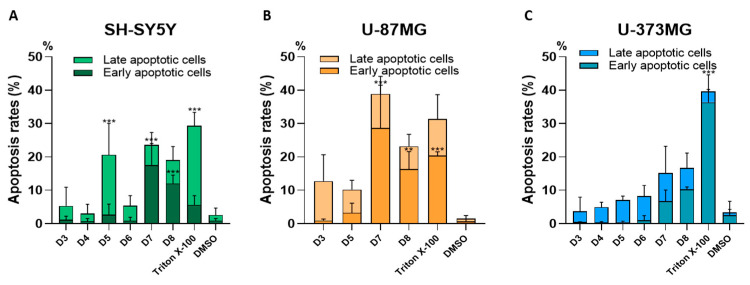
Effect of the sub-fractions D3–D8 (0.05 mg/mL) on cell death profile after 24 h of treatment of cell lines: (**A**) SH-SY5Y, (**B**) U-87MG and (**C**) U-373MG. Triton X-100, TX (1%) was used as a positive control. Data represent the means ± s.e.m. (standard error of mean) from at least three independent experiments (*n* = 3). ** *p* < 0.01 and *** *p* < 0.0001 compared to the respective control (cells treated with DMSO (vehicle) < 0.5%).

To investigate the contribution of apoptosis in suppressing cancer cell proliferation, the SH-SY5Y, U-87MG and U-373MG cell lines were treated with dichloromethane sub-fractions D3–D8 (0.05 mg/mL) for 24 h. Apoptosis was assessed using flow cytometry to detect phosphatidylserine externalization with FITC annexin V and cell membrane disruption with 7-AAD staining. The treatment with sub-fraction D5 significantly increased the percentage of SH-SY5Y cells in late apoptosis, as indicated by membrane disruption, which allowed for both annexin and 7-AAD staining (Figure 6A). Furthermore, in SH-SY5Y and U-87MG cells, sub-fractions D7 and D8 led to a significant rise in early apoptotic cells, as evidenced by increased phosphatidylserine externalization compared to untreated controls (Figure 6A,B). In contrast, no significant changes in apoptotic cells were observed in the U-373MG cell line treated with these sub-fractions (Figure 6C). It is worth noting that a high percentage of dead cells was observed in all three cell lines after 24 h of treatment. The FITC Annexin V assay/7AAD staining plots are shown in the Supplementary Material (Figure S2).

### 2.6. Sub-Fractions D7 and D8 Upregulated the Expression of Certain Apoptotic Proteins

**Figure 7:**
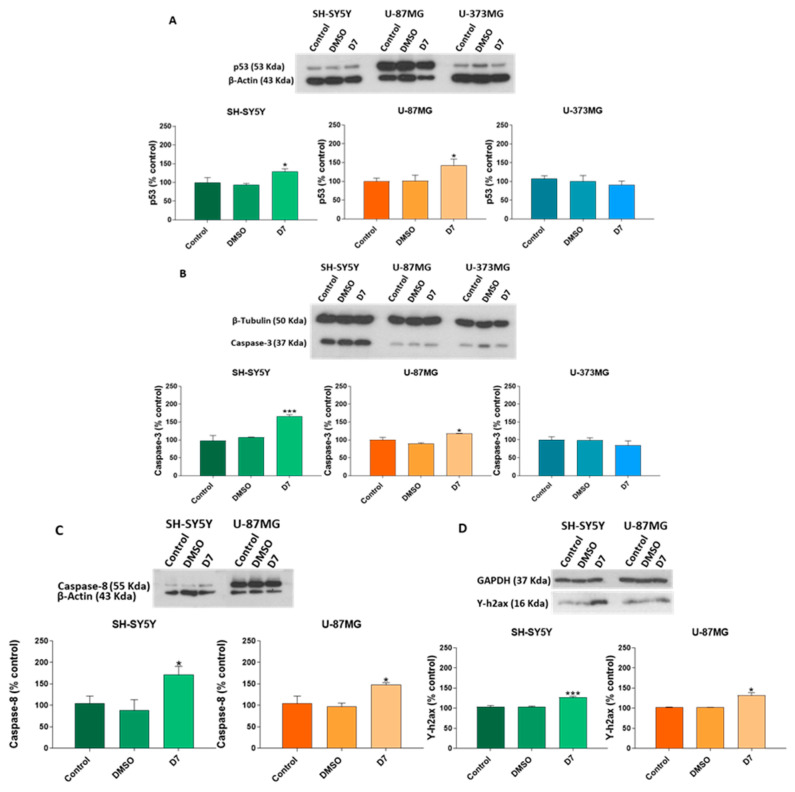
Effects of the sub-fraction D7 (0.05 mg/mL) on the expression of (**A**) p53 and (**B**) caspase-3 in SH-SY5Y, U-87MG and U-373MG cells lines and (**C**) caspase-8 and (**D**) ƴ-H2AX in SH-SY5Y and U-87MG cell lines after 24 h of treatment. DMSO: Cells treated with vehicle (<0.5%). Data represent the means ± s.e.m. (standard error of mean) from at least two independent experiments (*n* = 2). * *p* < 0.05 and *** *p* < 0.0001 compared to the control.

**Figure 8:**
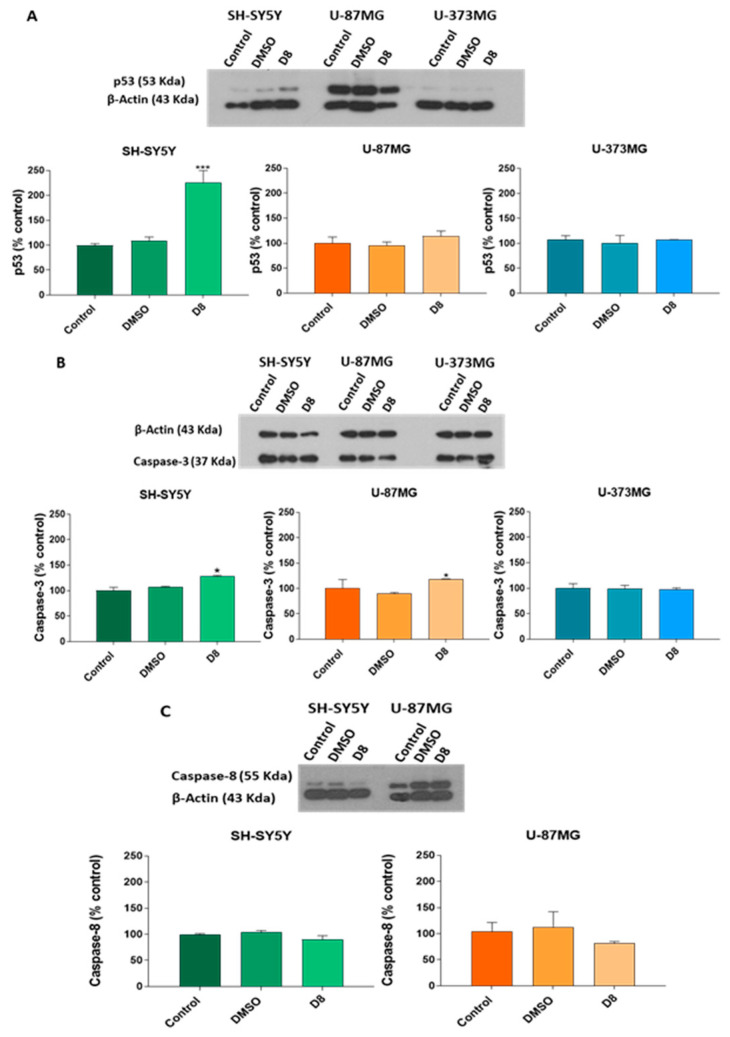
Effects of the sub-fraction D8 (0.05 mg/mL) on the expression of (**A**) p53 and (**B**) caspase-3 in SH-SY5Y, U-87MG and U-373MG cells lines and (**C**) caspase-8 in SH-SY5Y and U-87MG cell lines after 24 h of treatment. DMSO: Cells treated with vehicle (<0.5%). Data represent the means ± s.e.m. (standard error of mean) from at least two independent experiments (*n* = 2). * *p* < 0.05 and *** *p* < 0.0001 compared to the control.

The expression of apoptosis-related proteins was analyzed through western blot. Following 24 h of treatment with sub-fraction D7 (0.05 mg/mL), SH-SY5Y and U-87MG cells exhibited a significant upregulation in the expression levels of p53, caspase-3, caspase-8 and ƴ-H2AX (Figure 7A–D). Additionally, treatment with sub-fraction D8 resulted in increased caspase-3 levels, without affecting caspase-8 expression in both cell lines (Figure 8B,C). Sub-fraction D8 (0.05 mg/mL for 24 h) also increased p53 levels, specifically in SH-SY5Y cells. However, no changes in the expression of p53 or caspase-3 were observed in U-373MG cells following treatment with either sub-fraction D7 or D8 Figure 7A,B and Figure 8A,B).

### 2.7. Sub-Fractions D7 and D8 Induced Some Morphological Changes in SH-SY5Y, U-87MG and U-373MG Cells

**Figure 9:**
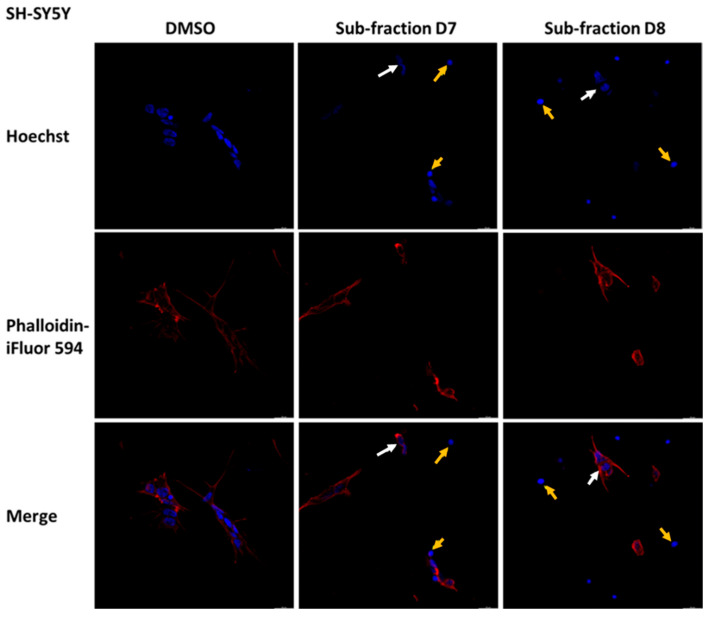
Effect of the sub-fractions D7 and D8 (0.05 mg/mL) on cell morphology after 24 h of treatment in SH-SY5Y cells using Hoescht (blue, chromatin status) and Phalloidin-iFluorTM 594 (red, cytoplasmatic traits). DMSO: Cells treated with vehicle (<0.5%). 20× magnification. Yellow arrows: apoptotic bodies; white arrow: alteration in nuclear morphology.

**Figure 10:**
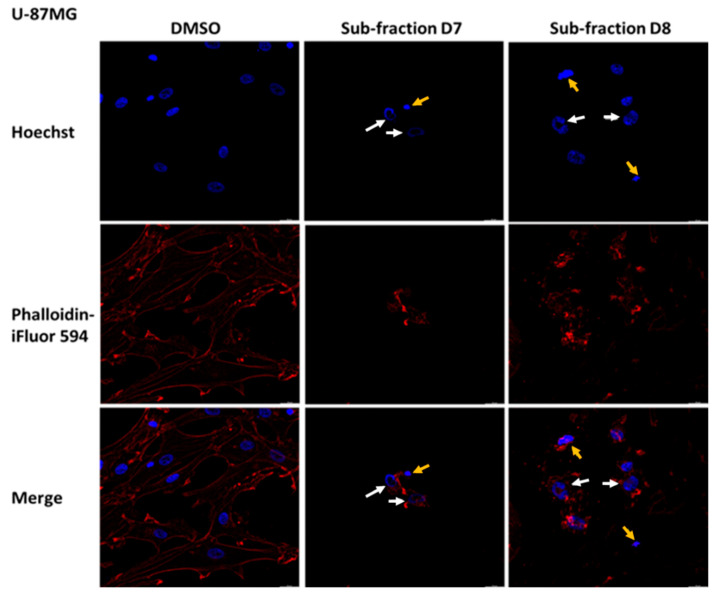
Effect of the sub-fractions D7 and D8 (0.05 mg/mL) on cell morphology after 24 h of treatment in U-87MG cells using Hoescht (blue, chromatin status) and Phalloidin-iFluor^TM^ 594 (red, cytoplasmatic traits). DMSO: Cells treated with vehicle (<0.5%). 20× magnification. Yellow arrows: apoptotic bodies; white arrow: alteration in nuclear morphology.

**Figure 11:**
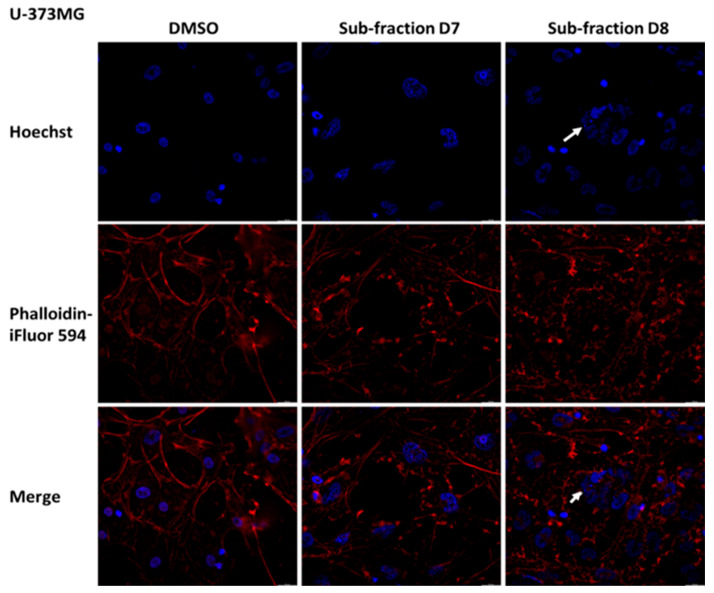
Effect of the sub-fractions D7 and D8 (0.05 mg/mL) on cell morphology after 24 h of treatment in U-373MG cells using Hoescht (blue, chromatin status) and Phalloidin-iFluorTM 594 (red, cytoplasmatic traits). DMSO: Cells treated with vehicle (<0.5%). 20× magnification. White arrow: alteration in nuclear morphology.

Considering the previous results, the effects of the sub-fractions D7 and D8 (0.05 mg/mL for 24 h) on the morphology of SH-SY5Y, U-87MG and U-373MG cells were explored. In SH-SY5Y cells treated with D7 and D8, Hoechst staining revealed alterations in nuclear morphology (white arrows) and the presence of apoptotic bodies (yellow arrows) (Figure 9). Additionally, in cells treated with D7, Phalloidin-iFluor^TM^ 594 staining revealed actin filament fragmentation. In U-87 MG cells, treatment with sub-fractions D7 and D8 induced apoptotic features not observed in untreated cells, including alterations in nuclear morphology and actin filament fragmentation (Figure 10). Similarly, in U-373MG cells, treatment with sub-fractions D7 and D8 also resulted in actin filament fragmentation, with sub-fraction D8 further revealing alterations in nuclear morphology (Figure 11).

### 2.8. Ultra-High-Pressure Liquid Chromatography–Mass Spectrometry (UHPLC-MS) Data Analysis of Sub-Fractions D7 and D8

In this study, UHPLC-MS data analysis was carried out by integrating different metabolomics platforms and software tools to facilitate compound annotation and generate a comprehensive metabolite profile potentially linked to the pharmacological activity. The UHPLC-QToF chromatograms of the sub-fractions D7 and D8 are provided in the Supplementary Material (Figures S3 and S4). After processing the raw data files, over 1800 metabolites were detected in each sub-fraction with the setup employed (Figures S5 and S6). Given that the pharmacological activity of the extracts is likely related to the most abundant analytes, compound annotation was focused on the most intense peaks/metabolites of sub-fractions D7 and D8. As a result of the UHPLC-MS data analysis carried out, 20 metabolites were tentatively identified in sub-fraction D7 and 15 in sub-fraction D8. The majority were flavonoids, including 14 isoflavones, 8 flavones, 5 flavanones and 3 flavonols. Additionally, one hydroxybenzaldehyde and two pterocarpans were identified. Fourteen of these metabolites had been previously identified in various *Ulex* species, while the remaining were known to be present in the Fabaceae family (Table 1 and Table 2).

In sub-fraction D7, the isoflavone isoprunetin was tentatively identified with [M-H]^−^ at 283.0613. Its fragmentation pattern presents ions at 268.0379, 240.0428 and 211.0402, which correspond to [M-H-CH3]^−^, [M-H-CH3-CO]^−^ and [M-2H-CH3-2CO]^−^, respectively, according to previously reported data [46] (Figure S7). Also, the isoflavone formononetin was tentatively identified with [M-H]^−^ at 267.0664, presenting fragment ions at 252.0427, corresponding to [M-H-CH3]^−^, and at 223.0400, corresponding to [M-H-CH3-CHO]^−^, characteristic ions of its fragmentation pattern [47] (Figure S8). These findings are remarkable, as isoprunetin accounted for 11.72% of the total chromatogram area, while formononetin represented 5.26%.

According to the isoflavonoid biosynthesis pathway, formononetin is closely related to other compounds such as daidzein, pseudobaptigenin, medicarpin and maackiain, all of which were also tentatively identified in this sub-fraction. Daidzein was tentatively identified with [M-H]^−^ at 253.0507, with fragment ions at 224.0479 and 209.0607 corresponding to [M-H-CHO]^−^ and [M-H-CO2]^−^, respectively [48]. Pseudobabtigenin was identified by its [M-H]^−^ at 281.0457, with fragment ions at 253.0506 and 135.0087, resulting from the retro-Diels–Alder reaction, a common fragmentation pathway of flavonoids [49]. Maackiain and medicarpin were identified by their [M-H]^−^ at 283.0613 and at 269.0820, respectively, with fragment ions compatible with their fragmentation’s patterns [50] (Figures S9–S12).

Additionally, several other metabolites of notable abundance in this sub-fraction were tentatively identified. Among them, the flavones quercetin-3,3’-dimethyl either with [M-H]^−^ at 329.0670 and licoflavone C with [M-H]^−^ at 337.1086 were assigned based on their fragmentation patterns and some relevant literature [51,52] (Figures S13 and S14).

In sub-fraction D8, the isoflavone genistein, representing 9.38% of the total chromatogram area, was identified with [M-H] ^−^ at 269.0459, along with fragment ions at 224.0479 and 133.0296, which are characteristic ions of its fragmentation pattern, as documented in the databases and the literature [48] (Figure S15). According to the isoflavonoid biosynthesis pathway, genistein is closely related to other compounds such as naringenin and apigenin, which were also tentatively identified in this sub-fraction. Naringenin was tentatively identified with [M-H]^−^ at 271.0613, displaying fragment ions at 151.0038 and 119.0503 corresponding to rings A and B of its structure, respectively [53] (Figure S16). Apigenin was tentatively identified with [M-H]^−^ at 269.0457, along with fragment ions compatible with its fragmentation pattern, as documented in the databases and the literature [54] (Figure S17).

Other metabolites of notable abundance in this sub-fraction were tentatively identified. The flavanone liquiritigenin, representing 4.60% of the total chromatogram area, was tentatively identified with [M-H]^−^ at 255.0666, along with fragment ions at 135.0089 and 119.0503, resulting from the retro-Diels–Alder reaction [47] (Figure S18). Additionally, kaempferol was tentatively identified with [M-H]^−^ at 285.0405, presenting fragment ions compatible with its fragmentation pattern, as documented in the databases and the literature [55] (Figure S19). Its direct precursor, dihydrokaempferol, was tentatively identified with [M-H]^−^ at 287.0563, along with fragment ions at 259.0613, corresponding to [M-H-CO]^−^, and 125.0244, a characteristic fragment ion for C-5 and C-7 dihydroxy-substituted flavanonols [56] (Figure S20).

The workflow employed does not differentiate between stereoisomers or closely related structural isomers. Multiple peaks with the same exact mass but distinct retention times were frequently observed in sub-fractions D7 and D8. The *m*/*z* values corresponding to the peaks with the same exact mass were the following: 269.045 (8 isomers), 271.061 (8 isomers), 283.061 (6 isomers), 285.040 (8 isomers), 299.056 (9 isomers), 329.067 (7 isomers), 337.108 (8 isomers), 353.103 (6 isomers) and 369.098 (10 isomers).

### 2.9. Gas Chromatography–Mass Spectrometry Analysis (GC-MS) of Sub-Fractions D7 and D8

GC-MS analysis identified 17 compounds in sub-fraction D7 and 9 in sub-fraction D8, with the majority being phenolic compounds. In sub-fraction D7, five flavonoids were detected, four of which had been previously tentatively identified through UHPLC-MS analysis: maackiain, formononetin, dinatin and pseudobabtigenin. Notably, dinatin and pseudobabtigenin had not been previously identified in *Ulex* species. Additionally, a flavonoid was detected at a retention time of 45.33 min and identified as 3,5,7-trimethoxyflavone (Figure S21 and Table 3).

In sub-fraction D8, three flavonoids were detected, two of which had been previously tentatively identified through UHPLC-MS analysis: naringenin and genistein. A flavonoid was also detected at 45.33 min and identified as 7-hydroxy-2-(3-hydroxyphenyl)-2,3-dihydro-4*H*-chromen-4-one (Figure S22 and Table 4).

### 2.10. Isolation, Purification and Identification of the Major Compounds of Sub-Fractions D7 and D8

Regarding the results for UHPLC-MS and GC-MS, the experimental methodology was adapted to isolate the major compounds in the sub-fractions.

**C1** (Isoprunetin, C_16_H_12_O_5_, 3.6 mg) was isolated from D7 as a colorless crystal. The ESI-FIA-TOF spectrum of this compound showed a peak at *m*/*z* 283.0612 corresponding to the molecular weight of the [M-H]^−^ ion of the tentatively identified compound, isoprunetin, in sub-fraction D7 (Figure S23). ^1^H and ^13^C NMR spectra of **C1** are provided in the Supplementary Material (Figure S24) and confirmed the chemical structure of isoprunetin.

ESI/MS (-) *m*/*z* 283.06 [M-H]^−^

^1^H NMR (600 MHz, CD_3_OD) δ 7.95 (1H, s), 7.32 (2H, d, *J* = 8.6 Hz), 6.81 (2H, d, *J* = 8.6 Hz), 6.42 (2H, d, *J* = 0.9), 3.88 (3H, s), in accordance with Boukaabache et al. [57]

^13^C NMR (151 MHz, CD_3_OD) δ 177.8, 164.9, 163.1, 161.5, 158.6, 152.6, 131.6, 127.0, 124.6, 116.1, 109.3, 97.5, 96.2, 56.5, in accordance with Fokialakis et al. [58].

**C2** (Genistein, C_15_H_10_O_5_, 5 mg) was isolated from D8 as a yellow powder. The ESI-FIA-TOF spectrum of this compound showed a peak at *m*/*z* 269.0454 corresponding to the molecular weight of the [M-H]^−^ ion of the tentatively identified compound, genistein, in sub-fraction D8 (Figure S25). ^1^H and ^13^C NMR spectra of **C2** are provided in the Supplementary Material (Figure S26) and confirmed the chemical structure of genistein.

ESI/MS (-) *m*/*z* 269.04 [M-H]^−^

^1^H NMR (600 MHz, CD_3_OD) δ 8.05 (1H, s), 7.37 (2H, d, *J* = 8.6 Hz), 6.84 (2H, d, *J* = 8.6 Hz), 6.34 (1H, d, *J* = 2.2 Hz), 6.22 (1H, d, *J* = 2.1 Hz), in accordance with Caligiani et al. [59].

^13^C NMR (151 MHz, CD_3_OD) δ 182.3, 166.3, 163.9, 159.8, 158.9, 154.8, 131.4, 124.8, 123.4, 116.3, 106.3, 100.3, 94.9, in accordance with He et al. [60].

### 2.11. Quantitative Analysis of Isoprunetin, Genistein and Naringenin in the Sub-Fractions D7 and D8 with HPLC-DAD

**Figure 12:**
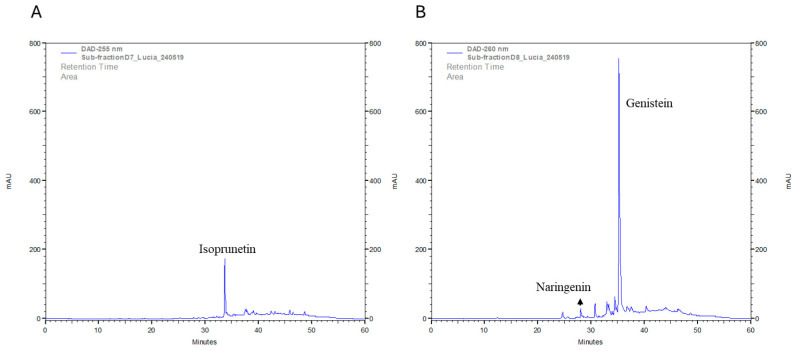
HPLC-DAD chromatograms at 255 nm of sub-fractions D7 (**A**) and D8 (**B**).

As shown in Figure 12A, isoprunetin was detected in the HPLC-DAD chromatogram of sub-fraction D7. It was found to be the most abundant compound in this sub-fraction, with a concentration of 142.91 µg/mL.

The HPLC-DAD chromatogram for sub-fraction D8 displayed one highly intense peak compared to the other peaks (Figure 12B). This peak was detected as genistein, with a concentration of 748.79 µg/mL. Additionally, naringenin was detected in this sub-fraction, with a concentration of 21.20 µg/mL.

However, both chromatograms revealed additional peaks beyond those for the studied compounds, suggesting the presence of other compounds. The retention time and absorbance values of the compounds were used to identify the peaks of interest in the chromatograms. HPLC chromatograms and UV spectra of the compounds are provided in the Supplementary Material (Figures S27–S29), and they were confirmed with previously reported data [46,61,62].

## 3. Discussion

This work investigated the potential activity of some sub-fractions of *U. gallii* in neuroblastoma and glioblastoma cell lines. Both cancers are prevalent, aggressive and associated with low survival rates. The primary objectives were to elucidate the mechanisms underlying the observed anti-proliferative effects of *U. gallii* sub-fractions and to explore the comprehensive metabolite profile potentially linked to their pharmacological activity.

The results obtained indicated that sub-fractions D3–D8 (0.1 mg/mL) significantly suppressed cell viability in the SH-SY5Y, U-87MG and U-373MG cell lines. These tumoral cells displayed greater susceptibility to the D3–D8 sub-fractions than the MRC-5 cells, a non-tumoral cell line, suggesting some selectivity of these sub-fractions towards the tumoral cells (Figure 1). Despite the interesting activity of the non-polar sub-fraction H3.3 suppressing cell viability in the same tumoral cells, it was excluded from further investigations due to its low solubility. Sub-fractions D3–D8 in SH-SY5Y and U-373MG cells, as well as D3, D5, D7 and D8 in U-87MG cells, were selected for further studies, as their decrease in cell viability exceeded that induced by topotecan (10 µM). Therefore, a depth investigation was carried out to determine whether the cell death mechanism induced by these sub-fractions was cytostatic (affecting only cell proliferation) or cytotoxic (leading to increased cell death). The selected concentration for these studies (0.05 mg/mL), intentionally lower than that employed in the viability assay, is close to the IC_50_ of the different sub-fractions. This concentration allows for the initiation of an apoptotic process without causing extensive cell death (Figure 2).

Plasma membrane rupture is a hallmark of necrotic cell death [63]. The LDH assay, which measures the release of this stable cytosolic enzyme upon cell membrane damage, showed that sub-fraction D8 significantly increased LDH release in U-373MG cells. This suggests that the necrotic process may play a role in its cytotoxicity (Figure 3).

Oxidative stress is implicated in various types of cell death due to an imbalance between ROS production and antioxidant defenses, leading to ROS accumulation and cellular damage. This can trigger different cell death pathways, such as necrosis and apoptosis. An increase in ROS levels has been associated with apoptosis in SH-SY5Y, U-87MG and U-373MG cells [64,65,66]. The treatment of U-87MG cells with sub-fractions D7 and D8 resulted in an increase in ROS levels, suggesting their potential involvement in an apoptotic process, further supported by the preservation of cell membrane integrity, as discussed below (Figure 4B).

Balancing cell cycle arrest is essential for preserving homeostasis and preventing cancer. In SH-SY5Y cells treated with sub-fractions D4 and D7, a significant increase in S-phase cells was observed, as well as in U-87MG cells treated with D3, D5, D7 and D8 (Figure 5A,B). Arrest in S-phase, where DNA replication occurs, could indicate that DNA breakage triggers the activation of DNA repair mechanisms, prolonging the duration of this phase. Additionally, DNA damage is closely associated with the induction of apoptosis [67,68,69]. In contrast, sub-fraction D8 induced G2/M phase arrest in SH-SY5Y cells (Figure 5A), whereas sub-fraction D5 caused G1 phase arrest in U-373MG cells (Figure 5C). G1 phase arrest is characteristic of antitumor agents that inhibit nucleotide synthesis, while G2/M phase arrest is associated with antitumor agents that affect microtubule dynamics [65,70,71,72].

Apoptosis is a form of cell death that occurs following cytotoxic drug treatment in various cancer types. Compounds that induce apoptosis are often non-toxic to healthy cells, highlighting their therapeutic potential in the search for new anticancer therapies [73]. Sub-fractions D7 and D8 showed a significant increase in early apoptosis in the SH-SY5Y and U-87MG cell lines (Figure 6A,B), suggesting tumor-suppressive properties via apoptosis induction. In contrast, sub-fraction D5 elevated late apoptosis in SH-SY5Y cells (Figure 6A) but was excluded from further analysis, as distinguishing between late apoptotic and secondary necrotic cells becomes challenging after plasma membrane permeabilization. Additionally, a high percentage of dead cells was observed in all three cell lines following 24 h of treatment, indicating that cell death may be triggered through additional mechanisms. This is a common occurrence with natural product extracts, which often comprise a complex mixture of bioactive compounds capable of modulating various cellular pathways and eliciting diverse responses simultaneously [74].

The effects of sub-fractions D7 and D8 on the expression of key proteins involved in apoptosis, such as the tumor suppressor p53, caspase-8 and caspase-3, were investigated. As a crucial regulator of the cell cycle and apoptosis, p53 plays a central role in detecting DNA damage and promoting repair while accelerating apoptosis through the activation of pro-apoptotic factors [75]. This function is well-documented in several plant-derived compounds, particularly flavonoids, which are known to induce p53 accumulation in various cancer cell types [76]. Sub-fraction D7 increased p53 expression in SH-SY5Y and U-87MG cells (Figure 7A), while sub-fraction D8 specifically elevated p53 expression in SH-SY5Y cells (Figure 8A), suggesting potential pro-apoptotic activity. Given p53′s dual role in promoting apoptosis or inducing growth arrest, additional pro-apoptotic proteins were examined to further elucidate the mechanism underlying the suppression of cell viability. Apoptosis occurs primarily through two main pathways: the extrinsic (death-receptor) and the intrinsic (mitochondrial) pathways. The extrinsic pathway is initiated through the activation of death receptors, leading to the recruitment and activation of caspase-8. In contrast, the intrinsic pathway is triggered by mitochondrial damage, resulting in the release of cytochrome c, which subsequently activates caspase-9. Both pathways converge downstream to activate executioner caspases, such as caspase-3 and caspase-7. Caspase-3 is a crucial executioner caspase, capable of cleaving numerous cellular substrates [77]. Sub-fraction D7 significantly increased the expression of caspase-8 and caspase-3 in both SH-SY5Y and U-87MG cells, suggesting the activation of the extrinsic apoptotic pathway (Figure 7B,C). In contrast, sub-fraction D8 specifically elevated caspase-3 levels without affecting caspase-8, indicating that it may activate a different apoptotic route. However, neither sub-fraction altered p53 or caspase-3 expression levels in U-373MG cells (Figure 7 and Figure 8), corresponding with the absence of observable apoptotic features in this cell line.

DNA double-strand break (DSB) is one of the most severe forms of DNA damage, with significant implications for cell death and genomic integrity. The phosphorylation of H2AX at Ser139, resulting in γ-H2AX, serves as an early marker of DSB [78]. To evaluate whether sub-fraction D7 induced DNA damage, the levels of phosphorylated histone (γ-H2AX) were assessed. Western blot analysis showed that subfraction D7 significantly elevated γ-H2AX expression in SH-SY5Y and U-87MG cells after 24 h of treatment (Figure 7D). This suggests that D7 may cause genomic instability, potentially triggering DNA damage responses that lead to apoptosis in these cell lines.

Membrane blebbing, chromatin condensation and the formation of apoptotic bodies are morphological changes characteristic of the apoptotic process [79]. The sub-fractions D7 and D8 elicited distinct nuclear changes consistent with these features in SH-SY5Y and U-87MG cells, indicating apoptosis and supporting the results obtained from these sub-fractions (Figure 9 and Figure 10). Additionally, in SH-SY5Y cells, sub-fraction D7 disrupted the actin cytoskeleton, and in U-87MG cells, both D7 and D8 did the same, a process linked to caspase activation that leads to the irreversible fragmentation of actin filaments [80]. In U-373 MG cells, only sub-fraction D8 affected the cell nucleus, while both D7 and D8 induced actin filament fragmentation (Figure 11).

Studies on the chemical composition of sub-fractions D7 and D8 were conducted as a complement to the previously published work [22]. In some cases, this new study yielded varying results for compound annotation. These discrepancies might arise from differences in the data and spectral libraries utilized by each database, as well as the presence of additional peaks unrelated to the compounds of interest, which may interfere with the interpretation of the respective mass spectra. However, in most cases, the different analysis converged on the same annotations. Herein, metabolomic analyses were performed using UHPLC-MS and GC-MS. While UHPLC-MS can identify a broader spectrum of compounds, it often provides limited certainty. Conversely, GC-MS offers high reproducibility in retention times and mass spectra, facilitating the identification of analytes through a comparison with a reference spectral database. Since some molecules are amenable to both GC-MS and UHPLC-MS approaches, using both methods provides complementary data on a wide range of analytes and allows for useful cross-validation.

To uncover a comprehensive profile of metabolites potentially related to the pharmacological activity of extracts, a non-targeted screening approach is desirable. The DIA-AIF technique used in UHPLC-MS analysis proved particularly useful for this purpose. UHPLC-MS analysis highlighted the chemical complexity of the studied sub-fractions, revealing the presence of over 1800 metabolites in each. To enhance the reliability of the annotation process, we adopted a workflow in which various platforms—each with distinct advantages and disadvantages—were used in a complementary manner for raw data processing and compound annotation. As a result of the procedure followed, 35 metabolites were identified in the two most active subfractions: 20 in sub-fraction D7 and 15 in sub-fraction D8. Most of the metabolites tentatively identified in these sub-fractions were flavonoids, highlighting *U. gallii* as a promising source of diverse flavonoids, particularly isoflavones and flavones. Multiple peaks with the same exact mass but distinct retention times were frequently observed in sub-fractions D7 and D8, likely indicating the presence of several closely related structural isomers with the same molecular formula. This observation suggests that the enzymes involved in flavonoid biosynthesis in *U. galli.* are largely promiscuous. In accordance with what is commonly seen in plant specialized metabolism, this enzyme promiscuity facilitates the formation of a “metabolic grid” that promotes the diversification of flavonoid chemical structures, potentially explaining the extensive variety of flavonoids found in *U. galli* [81].

In the absence of reference standards, the identification of metabolites using UHPLC-MS or GC-MS remains tentative. To confirm the identity of some metabolites and validate the analytical workflows employed, two tentatively identified analytes, isoprunetin and genistein, were isolated. Their structures were elucidated using NMR techniques and the spectral data were compared to those previously reported in the literature for these compounds [57,58,59,60]. Additionally, the quantification of the major compounds present in the sub-fractions was performed as discussed below.

The probable association between the abundance of flavonoids in the extracts and the observed pharmacological effects is consistent with previous research demonstrating that these compounds induce apoptotic mechanisms through caspase cascades in SH-SY5Y and U-87MG cells [82,83]. The observed effects, both in our study and others, may involve pathways beyond apoptosis, considering the inherent complexity of natural extracts, where numerous compounds can act synergistically, additively or antagonistically, leading to diverse cellular responses [74]. In sub-fraction D7, isoprunetin accounted for the highest percentage of the total chromatogram area at 11.72%, as confirmed by quantification analysis. However, cytotoxic activity for isoprunetin has only been reported to date in MCF-7 and A549 cells [84,85]. Formononetin was also tentatively identified in this sub-fraction, comprising 5.26% of the total chromatogram area, and its presence was later confirmed by GC-MS analysis. This compound is known to induce cell apoptosis through an intrinsic pathway involving Bax, Bcl-2 and caspase-3 proteins, and it promotes cell cycle arrest by regulating mediators such as cyclin A, cyclin B1 and cyclin D1 in various cancer cells [86]. Additionally, maackiain, also tentatively identified in this sub-fraction and subsequently confirmed by GC-MS analysis, has been reported to inhibit breast cancer progression by inducing apoptosis and to reduce the proliferation and migration of GBM cells [87,88]. Other flavonoids tentatively identified in sub-fraction D7, including 2,3-dehydrokievitone, medicarpin, daidzein and licoflavone C, have demonstrated the capability to induce apoptosis in various cancer cell lines [38,89,90,91].

In sub-fraction D8, the most abundant flavonoid identified was genistein. Genistein accounted for the highest percentage of the total chromatogram area at 9.38%, as confirmed by quantification analysis. It is well known as a tyrosine kinase inhibitor that has been reported to inhibit proliferation and induce apoptosis in different cancer cell lines, such as neuroblastoma and glioblastoma [92,93]. The second-most abundant flavonoid in this sub-fraction was tentatively identified as liquiritigenin, which has been shown to decrease viability in breast cancer MDAMB-231 and BT549 cell lines by increasing apoptosis and enhancing caspase-3 activity in these cells [94]. Naringenin was also tentatively identified and subsequently confirmed by GC-MS analysis, with quantification values revealing its high abundance in the sub-fraction. It has been highlighted for its antitumoral potential [95], exhibiting anti-tumor activity in U-87MG human glioblastoma cells and in xenograft mice models by inducing apoptosis and autophagy and activating the PI3K/Akt pathway [96]. Other flavonoids tentatively identified in sub-fraction D8, including kaempferol and apigenin, have also demonstrated the capability to induce apoptosis in various cancer cell lines [97,98].

The differing flavonoid compositions and their varying proportions in sub-fractions D7 and D8 may account for the distinct pharmacological effects observed between these fractions. However, with the current data, it is not possible to attribute the observed differences to specific compounds.

In conclusion, our findings demonstrated that sub-fractions D7 and D8 of *U. gallii* induce apoptosis in SH-SY5Y and U-87MG cells through distinct mechanisms, including ROS production in U-87MG cells. Despite the pronounced antiproliferative effects exhibited by these sub-fractions in U-373MG cells, no apoptotic hallmarks have been identified, and further studies are necessary to elucidate the underlying mechanism of action. Twenty metabolites were tentatively identified in sub-fraction D7, and fifteen were identified in sub-fraction D8. The majority were flavonoids, including 14 isoflavones, 8 flavones, 5 flavanones and 3 flavonols. The presence of these flavonoids may explain the pharmacological activity observed in sub-fractions D7 and D8.

## 4. Materials and Methods

### 4.1. Plant Material Collection, Obtention of Extracts and Sub-Fractions and Preparation of the Corresponding Solutions

Hexane, dichloromethane and methanol fractions from the methanol crude extract of *U. gallii* Planch. and the corresponding sub-fractions of hexane (H1, H3.1–3.6, H4), dichloromethane (D1–D8) and methanol (M1–M4) were previously obtained, as reported by Bada et al., 2023 [22]. A stock solution of each sub-fraction was prepared by dissolving the extract in DMSO (10 mg/mL), followed by stepwise dilution in culture medium to ensure a final DMSO concentration of ≤ 0.5%. The stepwise dilution was performed to avoid compound precipitation due to rapid concentration changes. To ensure solubility, samples were vortexed until completely dissolved and then centrifuged to check for any precipitate formation. Only sub-fraction H3.3 exhibited poorer solubility, requiring ultrasound and heating treatments. Once prepared, aliquots of the stock solution were stored at −20 °C, avoiding repeated freezing and thawing.

### 4.2. Cell Culture

Human neuroblastoma SH-SY5Y cells (American Type Culture Collection ATCC, Manassas, VA, USA) were cultured in a 1:1 mixture of Ham’s F12: Dulbecco’s Modified Eagle’s Medium (DMEM) supplemented with l-glutamine (2 mM), nonessential amino acids (1% *v*/*v*), fetal bovine serum (FBS, 10% *v*/*v*), penicillin (100 IU/mL) and streptomycin (100 µg/mL) (GIBCO, Invitrogen™, New York, USA) [99]. Human glioblastoma U-87MG and U-373MG cells (American Type Culture Collection ATCC) were cultured in DMEM Low glucose (1 g/L) supplemented with FBS (10% *v*/*v*), penicillin (100 IU/mL) and streptomycin (100 µg/mL) [100]. Human MRC-5 fibroblasts were cultured in MEM supplemented with FBS (10% *v*/*v*), penicillin (100 IU/mL) and streptomycin (100 µg/mL) [23]. The different cell lines were maintained under controlled conditions (a humid atmosphere with 5% CO_2_ at 37 °C) in an incubator (Binder CB150). The culture medium was replaced every 2 days, and after the cells reached 80–90% confluence, they were sub-cultured.

### 4.3. Determination of Cell Viability

Cell viability was measured in vitro in SH-SY5Y, U-87MG, U-373MG and MRC-5 cells using the 3-[4,5-dimethylthiazole-2-yl]-2,5-dimethyltetrazolium bromide (MTT) assay [101]. Cells were seeded in 96-well plates at 1 × 10^4^ cells/well and grown to confluence. Sub-fractions of *U. gallii* (0.1 mg/mL) dissolved in DMSO (<0.5% final concentration) were added to the medium. After 24 h of incubation, 10 µL of MTT (5 mg/mL) was added to each well and further incubated for 2 h at 37 °C. Afterwards, the culture medium was removed, and 100 µL of DMSO per well was added to solve the formazan crystals formed by the viable cells. Absorbance was quantified using a plate reader (Fluostar Optima^TM^, BMG LabTech, Offenburg, Germany) at a 540 nm wavelength. The anticancer molecule topotecan hydrochloride hydrate (10 µM) was used as a positive control. The viability (percentage) was calculated as [Absorbance (treatment)/Absorbance (negative control)] × 100. In addition, cell viability after treatment with sub-fractions D7 and D8 at a concentration of 0.05 mg/mL was evaluated using the trypan blue assay during the cell counting step of sample preparation for flow cytometry studies.

### 4.4. Assessment of Membrane Integrity—Lactate Dehydrogenase (LDH) Release Assay

The cells were seeded in 96-well plates as described above and grown to confluence. Subsequently, the cells were incubated with the D3–D8 sub-fractions (0.05 mg/mL) dissolved in DMSO (<0.5% final concentration) for 24 h. LDH was measured in the cell culture supernatant by monitoring the decrease in NADH during the conversion of pyruvate to lactate. The CyQUANT^TM^ LDH Cytotoxicity Assay Kit (Thermo Fisher Scientific, Waltham, MA, USA) was used according to the manufacturer’s instructions. Absorbance was measured at 490 and 680 nm using a plate reader (FluoStar Optima^TM^, BMG LabTech, Offenburg, Germany).

### 4.5. Intracellular Reactive Oxygen Species (ROS) Measurements

Intracellular ROS generation was measured in vitro using 2′,7′-dichlorodihydrofluorescein diacetate (H_2_DCF-DA). It is a cell-permeable indicator for ROS that is non-fluorescent until the acetate groups are removed by intracellular esterases and the oxidation of 2′,7′-dichlorodihydrofluorescein (H_2_DCF) occurs within the cell in the presence of hydrogen peroxide, yielding the fluorescent molecule dichlorofluorescein (DCF) [102]. Cells (5 × 10^5^ cells/well) were seeded in six-well plates and grown for 24 h. Subsequently, they were treated with the D3–D8 sub-fractions (0.05 mg/mL) dissolved in DMSO (<0.5% final concentration) for 24 h. Suspensions of each of them were prepared at a concentration of 200,000 cells/mL and incubated with an H_2_DCF-DA probe (5 µM) for 30 min at 37 °C. At least 1 × 10^5^ cells per sample were analyzed using the BD Accuri^TM^ flow cytometer (BD Biosciences, San Jose, CA, USA). H_2_O_2_ (100 µM) was used as a positive control. The number of H_2_DCFDA+ cells was calculated using the FlowJo software (v10.8.1, BD Biosciences, San Jose, CA, USA).

### 4.6. Cell Cycle Assay

Cells were seeded in six-well plates at a density of 5 × 10^5^ cells/well, grown to confluence and treated with the D3–D8 sub-fractions (0.05 mg/mL) dissolved in DMSO (<0.5% final concentration) for 24 h. Suspensions of each of them were prepared at a concentration of 200,000 cells/mL and fixed with MeOH for 30 min while shaking. Then, the cells were centrifuged and incubated in the dark with 100 µL of a propidium iodide solution (0.1 mg/mL) and 100 µL RNase (200 U/mL). At least 1 × 10^5^ cells per sample were analyzed using a BD Accuri^TM^ flow cytometer (BD Biosciences, San Jose, CA, USA). The percentages of cells in G0/G1, S and G2/M phases were calculated using the FlowJo software (v10.8.1, Biosciences).

### 4.7. Aminoactinomycin D (7AAD)/FITC Annexin V Assay

The apoptotic process was assessed using the FITC Annexin V Staining Kit (Tonbo Biosciences, San Diego, CA, USA). Cells were seeded in six-well plates (5 × 10^5^ cells/well), grown to confluence and treated with the D3–D8 sub-fractions (0.05 mg/mL) dissolved in DMSO (<0.5% final concentration) for 24 h. Suspensions of each treatment were prepared at a concentration of 220,000 cells in 100 µL of Annexin V Binding Buffer. FITC Annexin V conjugate (5 µL) and 7-AAD (5 µL) were added to each sample. After 15 min of incubation in the dark at room temperature, the samples were analyzed using the BD Accuri^TM^ flow cytometer (BD Biosciences, USA). Annexin V−/7-AAD− cells were considered viable, Annexin V+/7-AAD− corresponded to early apoptotic cells and Annexin V+/7-AAD+ were assigned to late apoptotic cells. The percentages of cells in each phase were calculated using the FlowJo software (v10.8.1, Biosciences). Triton X-100 (Sigma-Aldrich, St. Louis, MO, USA) (0.01%, 1 min) was used as positive control.

### 4.8. Western Blot

The cells were seeded in six-well plates at a density of 1.5 × 10^4^ cells/cm^2^ and grown to confluence. Then, SH-SY5Y, U87-MG and U-373MG cells were treated with the D7 and D8 sub-fractions (0.05 mg/mL) for 24 h at 37 °C. After 24 h of incubation, the cells were lysed according to our previous description [103]. Protein concentrations were determined using Bio-Rad Protein Assay Dye Reagent (Bio-Rad Laboratories GmbH, Hercules, CA, USA), according to the manufacturer’s protocol. Proteins (20 μg/well) were separated on 12% sodium dodecyl sulfate-polyacrylamide gel electrophoresis (SDS-PAGE) and transferred to polyvinylidene fluoride (PVDF) membranes (Bio-Rad Laboratories GmbH) using a Mini Trans-Blot^®^ Cell (Biorad Laboratories GmbH) for 1 h at 100 V. The membranes were blocked with 5% BSA at room temperature for 2 h. Afterwards, they were incubated overnight at 4 °C with primary monoclonal antibodies against caspase-3 (1:1000), caspase-8 (1:1000), p53 (1:1000) and ƴ-H2AX (1:500). Internal standards included antibodies against GAPDH (1:1000), β-actin (1:1000) and β-tubulin (1:5000). Secondary antibodies, anti-rabbit IgG (1:500) or anti-mouse IgG (1:5000) were used to incubate the membranes for 1 h at room temperature. Relative protein band densities were visualized using a chemiluminescent substrate (Pierce^TM^ ECL Western Blotting Substrate, Thermo Fisher Scientific, Waltham, MA, USA) and photographed (Fujimedical Super RX-N, Fujifilm, Tokyo, Japan). The images were analyzed using NIH ImageJ software 1.53t (Bethesda, MD, USA).

### 4.9. Morphological Analysis

Poly-d-lysine-coated coverslips were used to seed the cells at a density of 1 × 10^4^ cells/coverslip. After 24 h incubation, SH-SY5Y, U-87MG and U-373MG cells were treated with D7 and D8 sub-fractions (0.05 mg/mL) dissolved in DMSO (<0.5% final concentration) and further incubated for 24 h. Cells treated with DMSO (<0.5%) were used as a control. The cells were fixed with 4% *p*-formaldehyde for 15 min and then washed three times with PBS and permeabilized with 0.1% Triton X-100 for 5 min. After three additional washes with PBS, the cells were incubated with phalloidin conjugated with iFluor 594 at 0.5 μg/mL for 60 min. After three washes, the cells were incubated with Hoechst 33258 at 0.2 µg/mL for 15 min and then mounted with 90% glycerol/10% PBS on slides. Photos were taken with a Leica Thunder DM4 B at 20x magnification.

### 4.10. Ultra-High-Pressure Liquid Chromatography–Mass Spectrometry (UHPLC-MS) Analysis

UHPLC-MS analysis of the dichloromethane sub-fractions D7 and D8 was previously conducted using a UHPLC-QToF system as described by Bada et al., 2023 [22]. Bruker’s raw data files of sub-fractions D7 and D8 were analyzed again using MS-Dial software (https://systemsomicslab.github.io/compms/msdial/main.html; version 4.94, accessed on 3 April 2024) for peak detection, deconvolution, peak alignment, blank removal and peak matching against MS/MS public libraries. ProteoWizard software v3 was also used to convert Bruker’s raw data of sub-fractions D7 and D8 to mzXML files, which were subsequently processed with Mzmine software (https://mzmine.github.io/download.html; version 3.9.0, accessed on 3 April 2024) for peak detection, deconvolution and peak alignment, according to the parameters described in the manufacturer’s documentation. Compound annotation was conducted using several strategies. In general, the parameter settings were established as follows: MS1 and MS2 mass tolerances were set to 5 ppm and 10 ppm, respectively, and potential candidates were limited to those containing only C, H, O and N. In the first subset of experiments, peak information from MS-Dial (precursor *m*/*z*, retention time and experimental fragmentation patterns) was compared against free spectral database libraries using platforms such as mzCloud (https://www.mzcloud.org/, accessed on 14 May 2024), Reference Metabolome Database for Plants (RefMetaPlant; https://www.biosino.org/RefMetaDB/, accessed on 15 May 2024), Plant Metabolite Hub (Pmhub; https://pmhub.org.cn/#/, accessed on 16 May 2024) and MS-Dial. The spectral databases used included MassBank-EU, MassBank of North America (MoNA), ReSpect, Global Natural Products (GNPS), Fiehn HILIC, CASMI2016, metaboBASE, RIKEN PlaSMA, mzCloud and the specific experimental mass spectra collected in RefMetaPlant and PMhub. In the second subset of experiments, experimental precursor *m*/*z* values and fragmentation patterns obtained with MS-Dial were also compared with those estimated in silico from compounds present in natural product databases using two platforms, MS-Finder (https://systemsomicslab.github.io/compms/msfinder/main.html; version 3.61, accessed on 1 July 2024) and MetFrag (https://ipb-halle.github.io/MetFrag/; version 2.1, accessed on 2 July 2024). In the third subset of experiments, the data obtained for sub-fractions D7 and D8 from MZmine were exported to SIRIUS software (https://bio.informatik.uni-jena.de/software/sirius/; version 5.8.6, accessed on 9 April 2024) as an MGF file. SIRIUS can identify candidate molecular formulas and determine candidate structures by predicting their molecular fingerprints, which can be used to search in molecular structure databases for compound annotation. MS-Finder, MetFrag and SIRIUS enable the identification of small metabolites from experimental fragmentation data without relying on spectral libraries [104,105,106]. The data extraction and parameter setup were conducted in accordance with the manufacturer’s documentation.

The final selection of candidate compounds assigned to sub-fractions D7 and D8 was based on a manually weighted integration of all available information, including candidates with the highest similarity scores assigned by the metabolomic platforms when experimentally obtained MS-MS spectra were compared with those of authentic standards, the prioritization of MS-MS spectra from authentic standards over in silico generated spectra for comparisons, the analysis of compatibility with metabolic pathway maps, the manual interpretation of relevant MS-MS data such as the presence of some key fragments described in specific papers and the documented presence of candidates in the Ulex genus or the Fabaceae family.

### 4.11. Gas Chromatography–Mass Spectrometry (GC-MS) Analysis

The solutions of sub-fractions D7 and D8 (10 mg/mL in EtOAc), previously filtered, were applied to an Agilent 7890A gas chromatograph, coupled with an Agilent 5975C inert mass spectral detector (MSD) equipped with a triple axis detector and an Agilent 7693 autosampler (Agilent Technologies, Santa Clara, CA, USA). The transfer line, quadrupole and ion source temperatures were set at 290, 150 and 230 °C, respectively. Separation was performed using a ZB-Semivolatiles column (Phenomenex, Torrance, CA, USA). Helium (purity 99.99%) was used as the carrier gas at a flow rate of 1.0 mL/min. The GC oven temperature was programmed from 60 °C (held for 1 min) to 290 °C (held for 3 min), increasing at 5 °C/min. The total run time was 50 min, and the injection volume was 1 µL. The MSD operated in full scan (FS) acquisition mode, monitoring fragment mass/charge (*m*/*z*) between 89 and 800. The identification of compounds was performed by comparison (>75% match) between the experimental MS spectra and the MS spectra provided by a commercial spectral library database (National Institute of Standards and Technology, NIST, MS Search version 2.0).

### 4.12. Isolation, Purification and Identification of the Major Compounds 1–2 (C1–C2)

Sub-fraction D7 (512.2 mg in MeOH) was applied to a Biotage Sfär C18 (60 g) (Biotage, Uppsala, Sweden), column connected to a Biotage Select Flash instrument (Biotage, Uppsala, Sweden). Fractionation was carried out with a MeOH/H_2_O gradient (20–100%) and monitored by the UV absorbance at 254 nm. Seven UV-absorbing sub-fractions were obtained (D7F1–D7F7) (Table 5). Sub-fraction D7F7 was further purified using a Biotage Sfär C18 column (12 g) (Biotage, Uppsala, Sweden) under the same conditions, yielding eight UV-absorbing sub-fractions (D7F7F1-D7F7F8) (Table 5). Sub-fraction D7F7F6 was further purified using a ProStar Polaris HPLC system (Varian, Palo Alto, CA, USA) equipped with a Kinetex C18 100 Å (Phenomenex, Torrance, CA, USA) (150 × 21.2 mm, 5 µm) preparative column. The HPLC was operated at a flow rate of 10 mL/min, using a stepwise MeOH/H_2_O gradient (20–100%), with UV absorbance detection at 254 nm, resulting in sub-fractions D7F7F6S1–D7F7F6S5 (Table 5). D7F7F6S1 was identified as isoprunetin (compound 1, **C1**, 3.6 mg).

Sub-fraction D8 (33.5 mg in MeOH) was applied to a Biotage Sfär C18 (Biotage, Uppsala, Sweden, 12 g) column and fractioned using a MeOH/H_2_O gradient (25–100%), and UV absorbance detection at 260 nm. Fourteen UV-absorbing sub-fractions were obtained (D8F1-D8F14) (Table 6). The evaporation of sub-fraction D8F7 yielded genistein (compound 2, **C2**, 3.0 mg).

For the identification of **C1** and **C2**, 1D NMR spectroscopy (^1^H NMR and ^13^C NMR) was performed on a Bruker AVIII600 instrument (Bruker, Rheinstetten, Germany). CD_3_OD was used as a solvent and TMS was used as an internal reference. Mass spectra were acquired using a Bruker Impact II instrument (Bruker Corporation, Billerica, MA, USA in negative mode.

### 4.13. Quantitative Analysis of Isoprunetin, Genistein and Naringenin with HPLC-DAD

Sub-fractions D7 and D8 (1 mg/mL in 95% of H_2_O containing 0.1% TFA and 5% MeOH) were analyzed using a LaChrom Elite HPLC system (VWR-Hitachi, Tokyo, Japan) equipped with a L-2455 diode array detector and a Kinetex C18 100Å (150 × 4.6 mm) column. The gradient of mobile phases A (0.1% TFA in H_2_O) and B (MeOH) was used as follows: 5% B, 0–20 min; 90% B, 21–50 min; and 5% B, 51–60 min. The flow rate was 1 mL/min, and the injection volume was 10 µL. The oven temperature was set at 25 °C and absorbance was recorded at 230, 275, 300 and 330 nm. Quantification was performed using individual standard curves for each compound (isoprunetin and genistein, previously isolated as described above, and naringenin). Standard curves were made with eight different concentrations, each measured in triplicate, and the UV maxima for each compound were as follows: isoprunetin (255 nm), genistein (260 nm) and naringenin (290 nm) (Table 7). Chromatograms and UV spectra of the compounds are provided in the Supplementary Material (Figures S27–S29).

### 4.14. Statistical Analysis

The data are presented as the mean ± standard error of the mean (s.e.m.) of at least three independent experiments (*n* = 3). Statistical analyses were performed using one-way analysis of variance (ANOVA), followed by Dunnett’s multiple comparisons test (conducted with GraphPad Prism v.8.0.2).

## Figures and Tables

**Table 1:** Metabolites tentatively identified by UHPLC-MS data analysis in sub-fraction D7 (Rt = retention time; *m*/*z* = mass/charge ratio of the metabolite).

Peak	Rt (min)/Peak Area (%)	[M-H]^−^ (*m*/*z* Experimental)	*m*/*z* Error (ppm)	Main Fragments Ions (*m*/*z*)	Compound	Molecular Formula	Reference
10	4.40/2.28	121.0296	0.97	122.0329, 92.0268	4-Hydroxybenzaldehyde	C_7_H_6_O_2_	[30]
35	9.00/11.72	283.0613	0.33	284.0647, 271.0613, 268.0379, 240.0428, 211.0402	Isoprunetin	C_16_H_12_O_5_	[31]
38	9.70/3.97	253.0507	−0.09	254.0541, 224.0479, 211.0402, 209.0607	Daidzein	C_15_H_10_O_4_	[7]
40	10.40/3.28	283.0614	−0.20	284.0648, 268.0378	Biochanin A	C_16_H_12_O_5_	[32]
42	11.10/2.44	283.0613	0.25	284.0648, 268.0377, 240.0423, 211.0401, 149.0244	Maackiain	C_16_H_12_O_5_	[20]
46	13.10/2.34	269.0820	−0.08	270.0854, 253.0507	Medicarpin	C_16_H_14_O_4_	[33]
48	14.10/3.86	329.0670	0.49	330.0703, 327.0513, 299.0199, 271.0250	Quercetin-3,3′-dimethyl ether	C_17_H_14_O_7_	[34]
51	15.30/2.22	299.0562	−0.52	300.0597, 284.0326	Dinatin	C_16_H_12_O_6_	[35]
59	17.70/1.64	281.0457	0.13	282.0492, 253.0506, 135.0087	Pseudobabtigenin	C_16_H_10_O_5_	[36]
60	18.00/2.02	369.0983	0.71	370.1018, 297.0407	2,3-Dehydrokievitol	C_20_H_18_O_7_	[37]
61	18.40/5.26	267.0664	−0.05	268.0698, 252.0427, 223.0400, 195.0450	Formononetin	C_16_H_12_O_4_	[7]
66	19.90/3.66	353.1033	0.13	354.1068, 351.0879, 297.0407	2,3-Dehydrokievitone	C_20_H_18_O_6_	[38]
67	20.40/1.69	353.1034	0.13	354.1069	Licoflavonol	C_20_H_18_O_6_	[39]
69	20.80/1.66	351.0876	0.12	352.0910	Licoisoflavone B	C_20_H_16_O_6_	[8]
70	20.90/1.52	353.1034	0.88	354.1069, 335.0565, 281.0458	8-Prenylkaempferol	C_20_H_18_O_6_	[40]
82	24.30/4.36	337.1086	1.08	338.1120, 293.0458, 281.0457, 219.0666, 161.0244	Licoflavone C	C_20_H_18_O_5_	[37]
83	24.60/1.06	367.1191	0.38	368.1225	Gancaonin N	C_21_H_20_O_6_	[37]
86	25.20/4.55	337.1084	1.25	338.1118, 112.9857	Wighteone	C_20_H_18_O_5_	[41]
93	26.90/2.59	335.0929	1.07	336.0964, 320.0691	Limonianin	C_20_H_16_O_5_	[8]
103	30.40/3.95	405.1710	1.02	406.1744	Isolupabigenin	C_25_H_26_O_5_	[9]

**Table 2:** Metabolites tentatively identified by UHPLC-MS data analysis of sub-fraction D8 (Rt = retention time; *m*/*z* = mass/charge ratio of the metabolite).

Peak	Rt (min)/Peak Area (%)	[M-H]^−^ (*m*/*z* Experimental)	*m*/*z* Error (ppm)	Main Fragments Ions (*m*/*z*)	Compound	Molecular Formula	Reference
19	6.80/0.86	271.0613	0.18	272.0647, 253.0506, 243.0663, 135.0089, 91.0190	Butin	C_15_H_12_O_5_	[42]
23	7.50/1.32	299.0562	0.20	300.0597, 284.0323, 255.0663	Isokaempferide	C_16_H_12_O_6_	[43]
24	7.60/2.31	287.0563	−0.14	288.0597, 269.0456, 259.0613, 177.0557, 125.0244	Dihydrokaempferol	C_15_H_12_O_6_	[7]
31	9.40/0.89	417.1193	0.27	418.1227, 329.0669, 255.0663, 135.0088	Liquiritin	C_21_H_12_O_9_	[7]
32	9.60/0.95	285.0405	0.17	286.0440, 217.0507, 175.0401	Luteolin	C_15_H_10_O_6_	[7]
35	10.10/2.06	285.0405	−0.15	286.0439, 257.0455, 151.0038	Kaempferol	C_15_H_10_O_6_	[44]
39	11.10/0.91	329.0669	0.50	330.0703	Quercetin-3,3′-dimethyl ether	C_17_H_14_O_7_	[34]
42	11.80/1.85	313.0719	−0.50	314.0754, 285.0406	Onogenin	C_17_H_14_O_6_	[8]
47	13.30/2.41	271.0613	0.65	272.0648, 163.0038, 151.0038, 119.0503	Naringenin	C_15_H_12_O_5_	[7]
50	14.30/9.38	269.0459	1.20	270.0491, 241.0506, 225.0554, 201.0559, 181.0659, 159.0452, 133.0296, 107.0141	Genistein	C_15_H_10_O_5_	[7]
54	15.80/1.79	269.0457	0.70	270.0492, 117.0347, 107.0141	Apigenin	C_15_H_10_O_5_	[44]
62	18.80/4.60	255.0666	0.18	256.0698, 135.0088	Liquiritigenin	C_15_H_12_O_4_	[7]
64	19.20/0.84	337.0721	0.44	338.0753, 283.0613	Neotenone	C_19_H_14_O_6_	[44]
67	19.70/0.93	369.0981	0.25	370.1015, 301.1083	Gancaonin P	C_20_H_18_O_7_	[45]
68	19.90/0.90	353.1033	0.25	354.1068, 351.0879, 297.0408	2,3-Dehydrokievitone	C_20_H_18_O_6_	[38]

**Table 3:** Metabolites tentatively identified by GC-MS data analysis in the sub-fraction D7 (Rt = Retention time).

Compounds in Sub-Fraction D7	Molecular Formula	Rt (min)
l-leucine	C_21_H_41_NO_4_	10.57
4-Hydroxybenzaldehyde	C_7_H_6_O_2_	15.32
Vanillin	C_8_H_8_O_3_	16.10
Isoeugenol	C_10_H_12_O_2_	17.40
d-Allose	C_6_H_12_O_6_	18.86
β-d-Glucopyranoside	C_7_H_14_O_6_	21.06
4-((1*E*)-3-Hydroxy-1-propenyl)-2-methoxyphenol	C_10_H_12_O_3_	22.54
4-Hydroxy-2-methoxycinnamaldehyde	C_10_H_10_O_3_	23.93
4-((1*E*)-3-Hydroxy-1-propenyl)-2-methoxyphenol	C_10_H_12_O_3_	24.11
Benzoic acid	C_9_H_10_O_5_	25.64
3,5-Dimethoxy-4-hydroxycinnamaldehyde	C_11_H_12_O_4_	28.93
Maackiain	C_16_H_12_O_5_	40.78
Formononetin	C_16_H_12_O_4_	42.85
Dinatin	C_16_H_12_O_6_	43.90
Pseudobabtigenin	C_16_H_10_O_5_	44.63
(2*Z*)-6-hydroxy-2-[(4-hydroxy-3-methoxyphenyl) methylidene]-1-benzofuran-3-one	C_16_H_12_O_5_	44.87
3,5,7-Trimethoxyflavone	C_18_H_16_O_5_	45.33

**Table 4:** Metabolites tentatively identified by *G*C-MS data analysis in sub-fraction D8 (Rt = Retention time).

Compounds in Sub-Fraction D8	Molecular Formula	Rt (min)
l-Leucine	C_16_H_31_NO_4_	10.57
d-Allose	C_6_H_12_O_6_	18.57
2-*cis*-9-Octadecenyloxyethanol	C_20_H_40_O_2_	26.16
Palmitic acid	C_17_H_34_O_2_	28.73
Stearic acid	C_18_H_36_O_2_	32.39
Dehydroabietic acid	C_20_H_28_O_2_	36.84
Naringenin	C_15_H_12_O_5_	43.07
7-Hydroxy-2-(3-hydroxyphenyl)-2,3-dihydro-4*H*-chromen-4-one	C_15_H_12_O_4_	44.05
Genistein	C_15_H_10_O_5_	44.66

**Table 5:** Quantities obtained and percentage yields of sub-fractions D7F1–D7F7.

Sub-Fractions	Sub-Fraction Weight (g)	Yield (%)
D7F1	0.076	14.84
D7F2	0.037	7.23
D7F3	0.100	19.53
D7F4	0.061	11.91
D7F5	0.036	7.03
D7F6	0.077	15.04
D7F7	0.086	16.80
D7F7F1	0.005	5.81
D7F7F2	0.006	6.98
D7F7F3	0.003	3.49
D7F7F4	0.004	4.65
D7F7F5	0.003	3.48
D7F7F6	0.010	11.63
D7F7F7	0.003	3.49
D7F7F8	0.003	3.49
D7F7F6S1	0.004	40.00
D7F7F6S2	0.002	20.00
D7F7F6S3	0.002	20.00
D7F7F6S4	0.001	10.00
D7F7F6S5	0.001	10.00

**Table 6:** Quantities obtained and percentage yields of sub-fractions D8F1–D8F14.

Sub-Fractions	Sub-Fraction Weight (g)	Yield (%)
D8F1	0.001	3.03
D8F2	0.001	3.03
D8F3	0.001	3.03
D8F4	0.001	3.03
D8F5	0.002	6.07
D8F6	0.003	9.10
D8F7	0.003	9.10
D8F8	0.002	6.07
D8F9	0.002	6.07
D8F10	0.003	9.10
D8F11	0.003	9.10
D8F12	0.002	6.07
D8F13	0.003	9.10
D8F14	0.002	6.07

**Table 7:** Calibration curve, limit of detection (LOD) and limit of quantification (LOQ) of isoprunetin, genistein and naringenin. ^a^ Eight different concentrations were used. ^b^ LOD, 3.3 × standard deviation of the ƴ-intercepts of the regression line/slope of the regression line (Ϭ/S). ^c^ LOQ, 10 × standard deviation of the ƴ-intercepts of the regression line/slope of the regression line (Ϭ/S).

Compound	Calibration curve	R^2^	Concentration Range (µg/mL) ^a^	LOD ^b^ (µg/mL)	LOQ ^c^ (µg/mL)
Isoprunetin (**C1**)	y = 1.1711x − 5.0918	0.9938	1–200	7.97	24.15
Genistein (**C2**)	y = 1.0113x − 2.6901	0.9967	1–200	5.80	15.58
Naringenin	y = 0.9926x + 0.0754	0.9985	1–200	3.90	11.83

## Data Availability

Data are contained within the article and Supplementary Materials.

## Supplementary Materials

The following supporting information can be downloaded at: https://www.mdpi.com/article/10.3390/molecules30040972/s1. Figure S1. Representative cell cycle histograms of the cell lines: A) SH-SY5Y, B) U-87MG and C) U-373MG, after 24 h treatment with sub-fractions D3-D8 (0.05 mg/mL) and DMSO (vehicle) < 0.5%; Figure S2. Representative dot-plots of 7AAD staining/FITC Annexin V assay results of the cell lines: A) SH-SY5Y, B) U-87MG and C) U-373MG, after 24 h treatment with sub-fractions D3-D8 (0.05 mg/mL), Triton X-100 (1%), and DMSO (vehicle) < 0.5%; Figure S3. UHPLC-QToF chromatogram of sub-fraction D7 of *U. gallii*; Figure S4. UHPLC-QToF chromatogram of sub-fraction D8 of *U. gallii*; Figure S5. Metabolites detected in D7; Figure S6. Metabolites detected in D8; Figure S7. Mass spectrum corresponding to isoprunetin (peak 35, Rt: 9.00 min) in sub-fraction D7; Figure S8. Mass spectrum corresponding to formononetin (peak 61, Rt: 18.40 min) in sub-fraction D7; Figure S9. Mass spectrum corresponding to daidzein (peak 38, Rt: 9.70 min) in sub-fraction D7; Figure S10. Mass spectrum corresponding to pseudobabtigenin (peak 59, Rt: 17.70 min) in sub-fraction D7; Figure S11. Mass spectrum corresponding to maackiain (peak 42, Rt: 11.10 min) in sub-fraction D7; Figure S12. Mass spectrum corresponding to medicarpin (peak 46, Rt: 13.10 min) in sub-fraction D7; Figure S13. Mass spectrum corresponding to quercetin-3,3-dimethyl ether (peak 48, Rt: 14.10 min) in sub-fraction D7; Figure S14. Mass spectrum corresponding to licoflavone C (peak 82, Rt: 24.30 min) in sub-fraction D7; Figure S15. Mass spectrum corresponding to genistein (peak 50, Rt: 14.30 min) in sub-fraction D8; Figure S16. Mass spectrum corresponding to naringenin (peak 47, Rt: 13.30 min) in sub-fraction D8; Figure S17. Mass spectrum corresponding to apigenin (peak 54, Rt: 15.80 min) in sub-fraction D8; Figure S18. Mass spectrum corresponding to liquiritigenin (peak 62, Rt: 18.80 min) in sub-fraction D8; Figure S19. Mass spectrum corresponding to kaempferol (peak 35, Rt: 10.10 min) in sub-fraction D8; Figure S20. Mass spectrum corresponding to dihydrokaempferol (peak 24, Rt: 7.60 min) in sub-fraction D8; Figure S21. GC-MS chromatogram of sub-fraction D7 of *U. gallii*; Figure S22. GC-MS chromatogram of sub-fraction D8 of *U. gallii*; Figure S23. ESI-FIA-TOF spectrum of isoprunetin (C1); Figure S24. ^1^H NMR(A) and ^13^C NMR (B) spectra of C1 in CD_3_OD; Figure S25. ESI-FIA-TOF spectrum of genistein (C2); Figure S26. ^1^H NMR (A) and ^13^C NMR (B) spectra of C2 in CD_3_OD; Figure S27. HPLC chromatogram at 255 nm (left) and UV spectrum (right) of isoprunetin (C1); Figure S28. HPLC chromatogram at 260 nm (left) and UV spectrum (right) of genistein (C2); Figure S29. HPLC chromatogram at 290 nm (left) and UV spectrum (right) of naringenin.
