# Supplementary material for: Antitumor Activity, Mechanisms of Action and Phytochemical Profiling of Sub-Fractions Obtained from Ulex gallii Planch. (Fabaceae): A Medicinal Plant from Galicia (Spain)
Source: Molecules. 2025 Feb 19;30(4):972. doi: 10.3390/molecules30040972 (PMC11858089; doi:10.3390/molecules30040972)
Supplement: Supplementary file 1 [file molecules-30-00972-s001.zip › molecules-3448422-supplementary.pdf]

molecules

MDPI

Supplementary Material

Antitumor Activity, Mechanisms of Action and Phytochemical Profiling of Sub-fractions Obtained from *Ulex gallii* Planch. (Fabaceae): a Medicinal Plant from Galicia (Spain)

Lucía Bada[1,2], Hussain Shakeel Butt[3], Elías Quezada[2], Aitor Picos[1,2], Helle Wangensteen[3], Kari Tvete Ingjerdingen[3], José Gil-Longo[2], and Dolores Viña [1,2]*

[1] Group of Pharmacology of Chronic Diseases (CD Pharma), Molecular Medicine and Chronic Diseases Research Centre (CIMUS), Universidade de Santiago de Compostela, 15782 Santiago de Compostela, Spain; lucia.bada@rai.usc.es; aitor.picos@rai.usc.es

[2] Department of Pharmacology, Pharmacy and Pharmaceutical Technology, Faculty of Pharmacy, Universidade de Santiago de Compostela, 15782 Santiago de Compostela, Spain; elias.quezada@usc.es; jose.gil.longo@usc.es

[3] Section for Pharmaceutical Chemistry, Department of Pharmacy, University of Oslo, 0316 Oslo, Norway; hsb95@hotmail.no; helle.wangensteen@farmasi.uio.no; k.t.inngjerdingen@farmasi.uio.no

* Correspondence: mdolores.vina@usc.es; Tel.: +34 881 815 424

2. Results

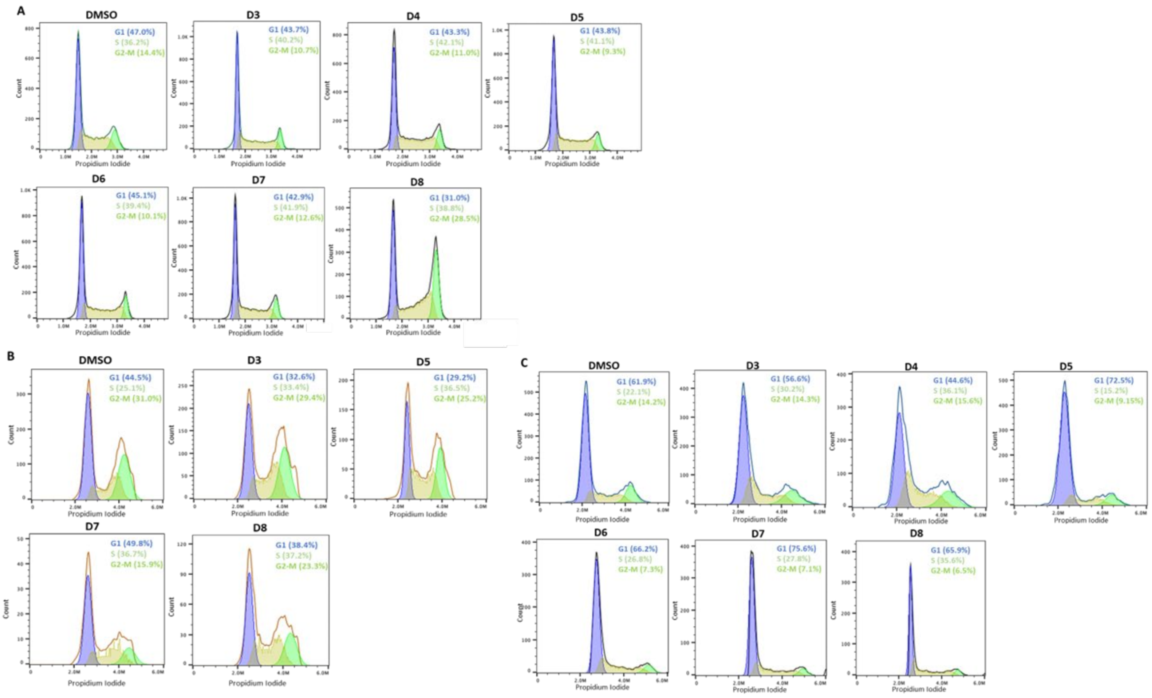

Figure S1. Representative cell cycle histograms of the cell lines: A) SH-SY5Y, B) U-87MG and C) U-373MG, after 24 h treatment with sub-fractions D3-D8 (0.05 mg/mL) and DMSO (vehicle) < 0.5%.

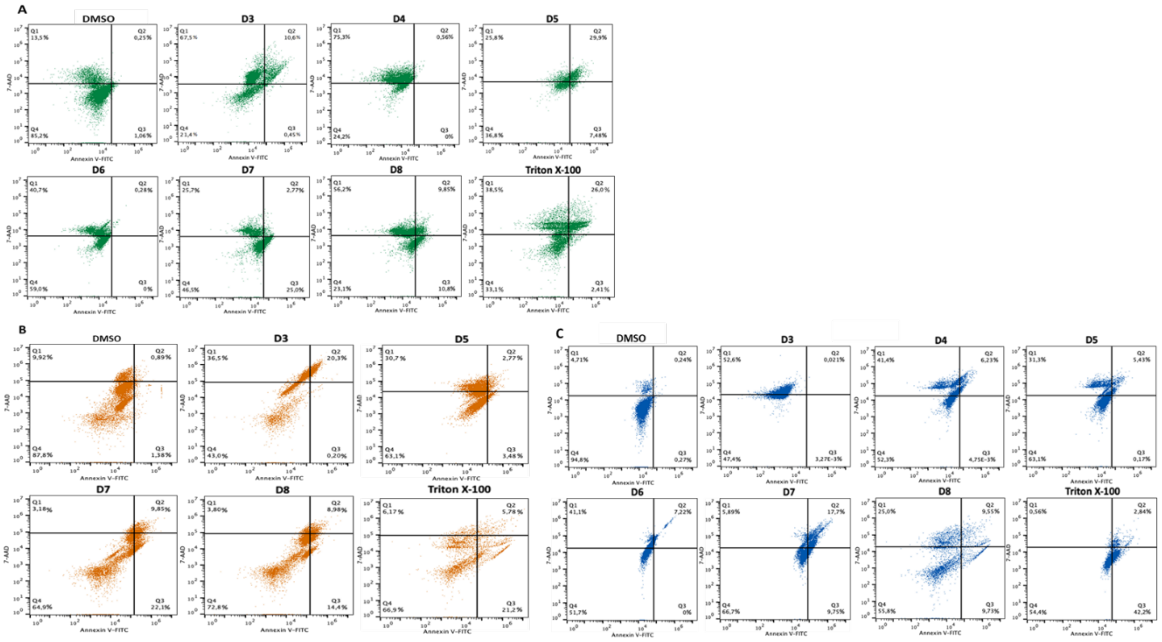

Figure S2. Representative dot-plots of 7AAD staining/FITC Annexin V assay results of the cell lines: A) SH-SY5Y, B) U-87MG and C) U-373MG, after 24 h treatment with sub-fractions D3-D8 (0.05 mg/mL), Triton X-100 (1%), and DMSO (vehicle) < 0.5%.

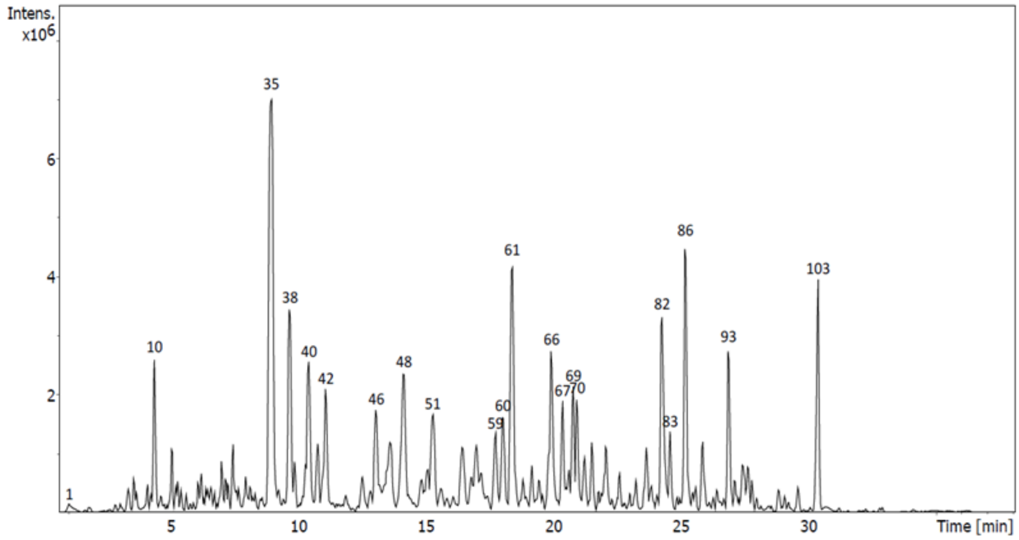

Figure S3. UHPLC-QToF chromatogram of sub-fraction D7 of *U. gallii* [1].

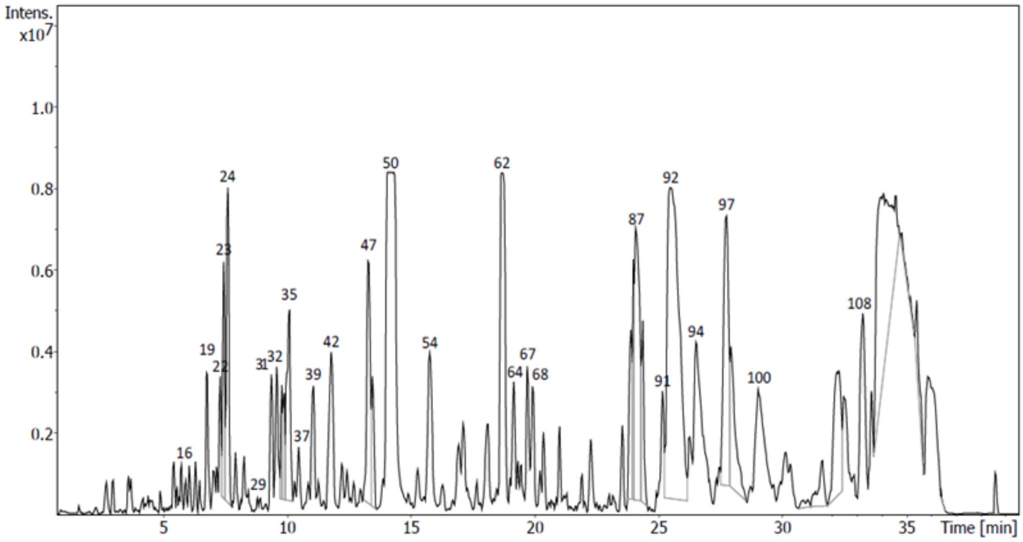

Figure S4. UHPLC-QToF chromatogram of sub-fraction D8 of *U. gallii* [1].

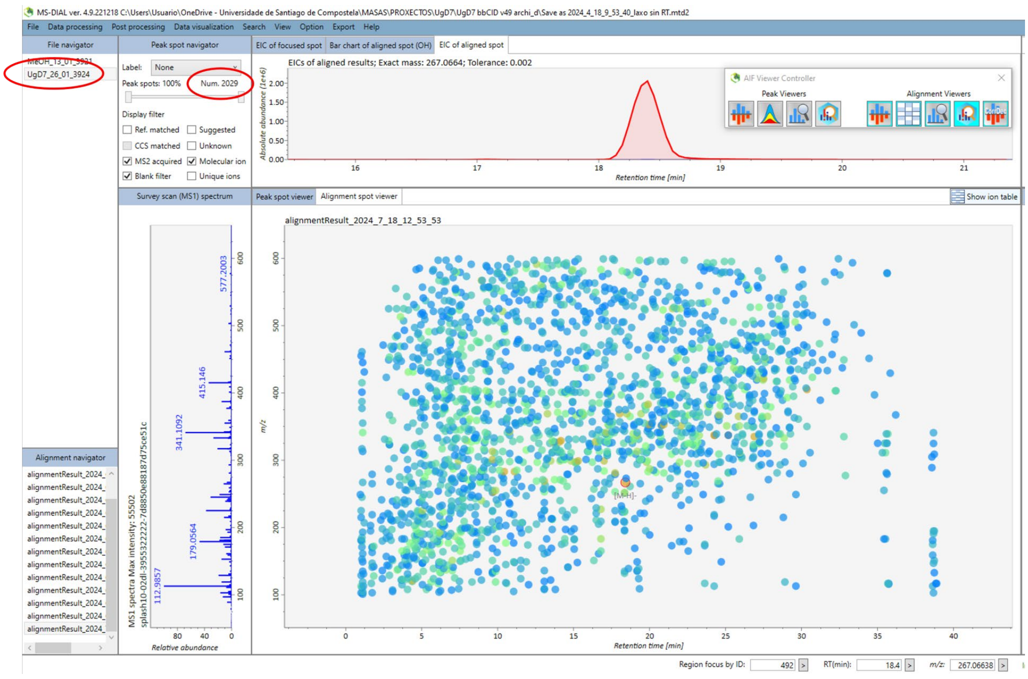

Figure S5. Metabolites detected in D7.

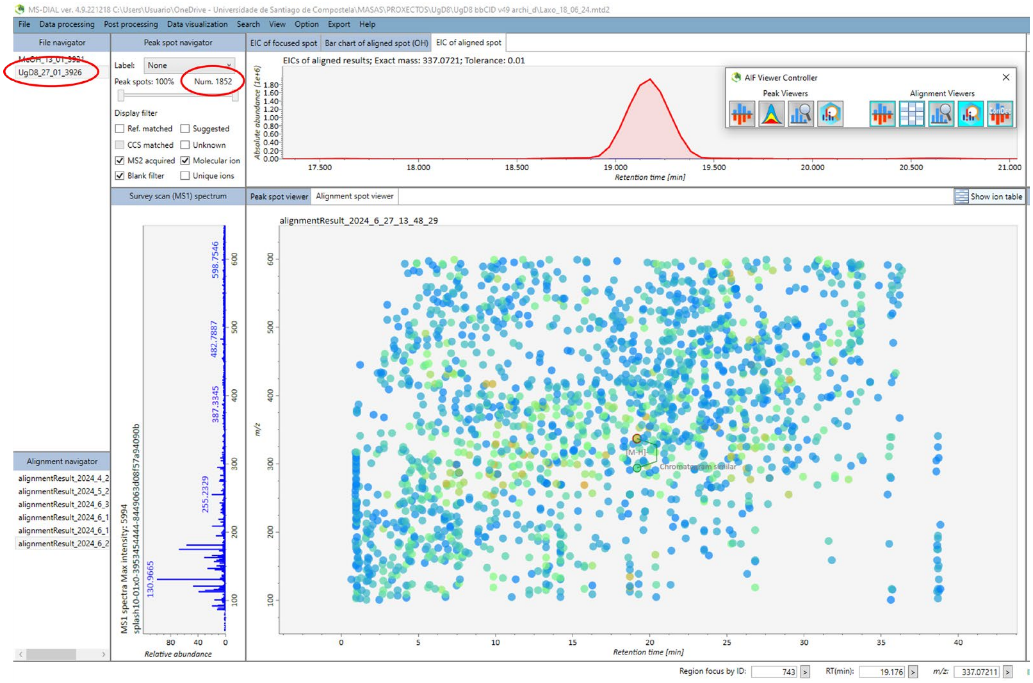

Figure S6. Metabolites detected in D8.

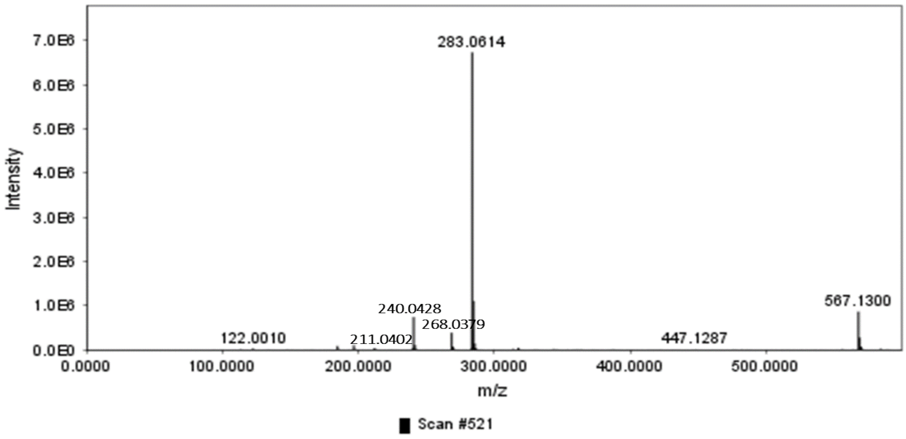

Figure S7. Mass spectrum corresponding to isoprunetin (peak 35, Rt: 9.00 min) in sub-fraction D7.

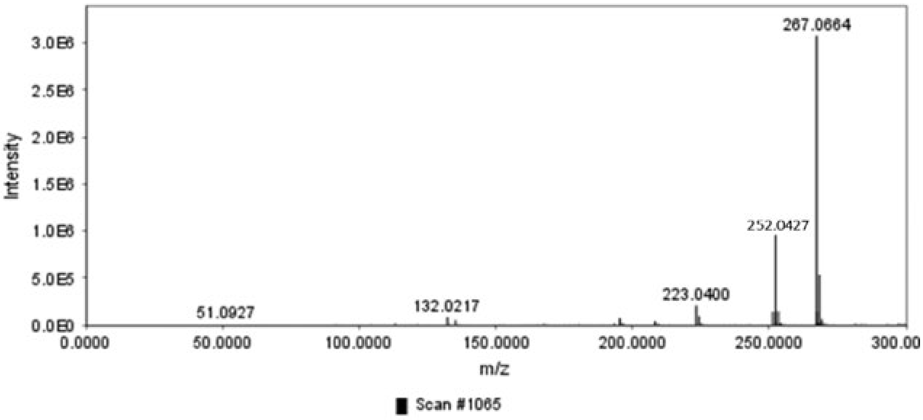

Figure S8. Mass spectrum corresponding to formononetin (peak 61, Rt: 18.40 min) in sub-fraction D7.

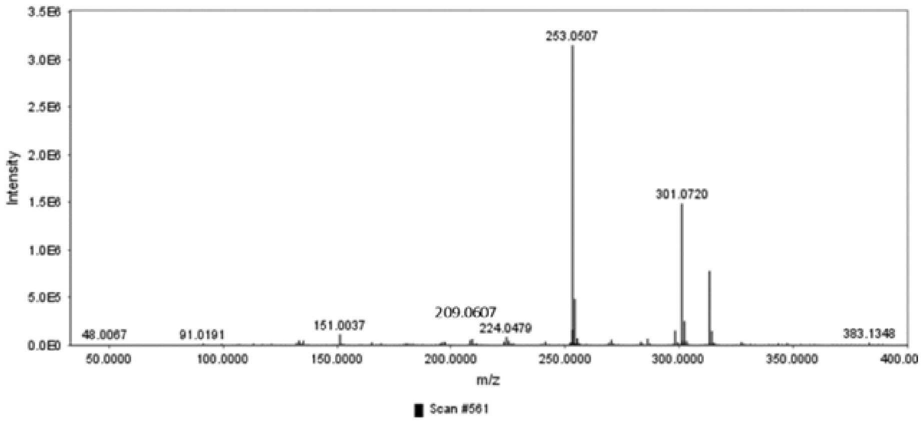

Figure S9. Mass spectrum corresponding to daidzein (peak 38, Rt: 9.70 min) in sub-fraction D7.

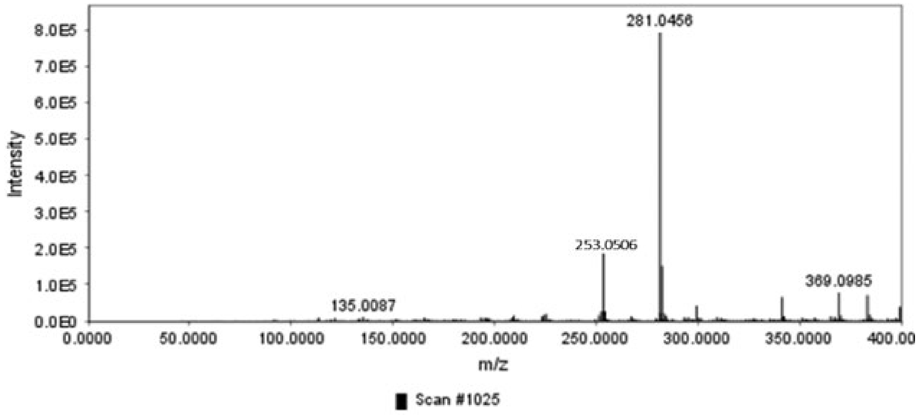

Figure S10. Mass spectrum corresponding to pseudobabtigenin (peak 59, Rt: 17.70 min) in sub-fraction D7.

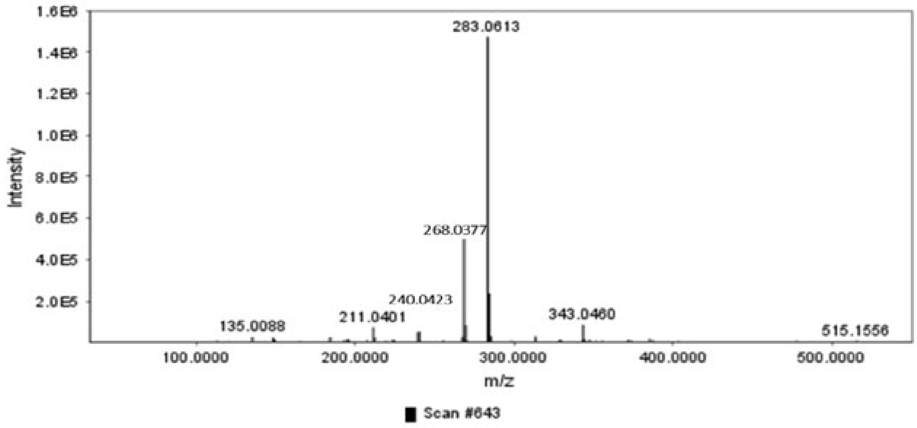

Figure S11. Mass spectrum corresponding to maackiain (peak 42, Rt: 11.10 min) in sub-fraction D7.

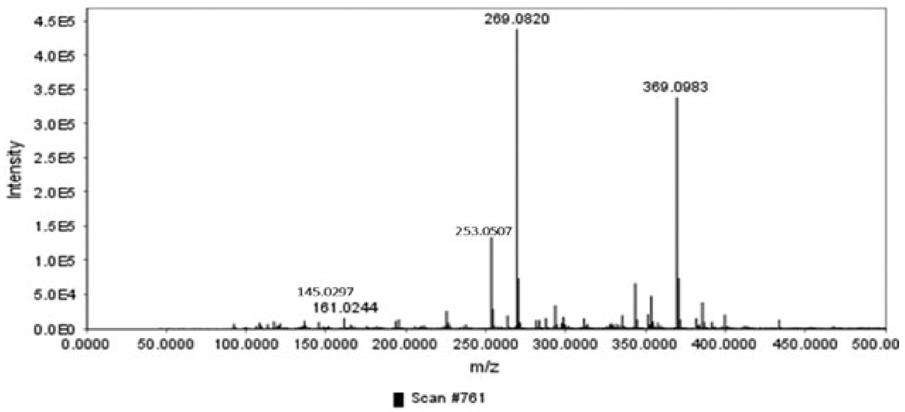

Figure S12. Mass spectrum corresponding to medicarpin (peak 46, Rt: 13.10 min) in sub-fraction D7.

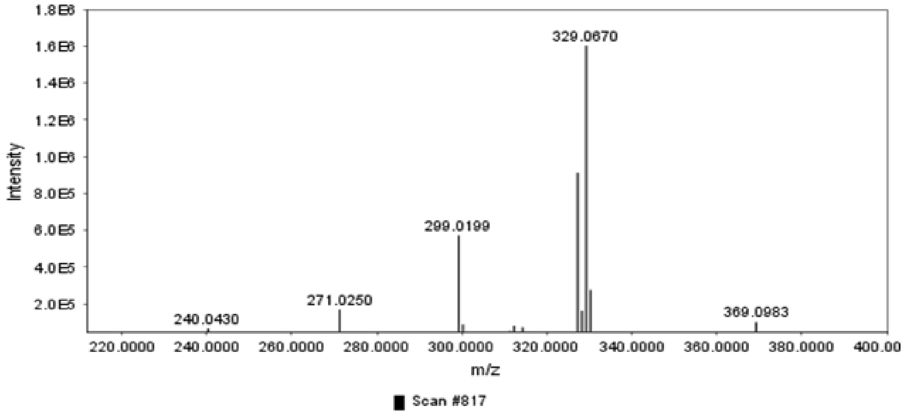

Figure S13. Mass spectrum corresponding to quercetin-3,3-dimethyl ether (peak 48, Rt: 14.10 min) in sub-fraction D7.

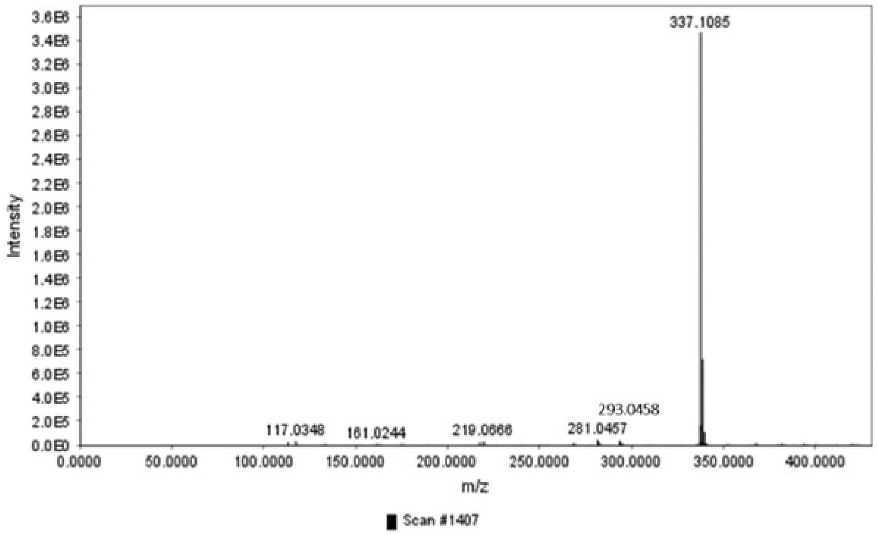

Figure S14. Mass spectrum corresponding to licoflavone C (peak 82, Rt: 24.30 min) in sub-fraction D7.

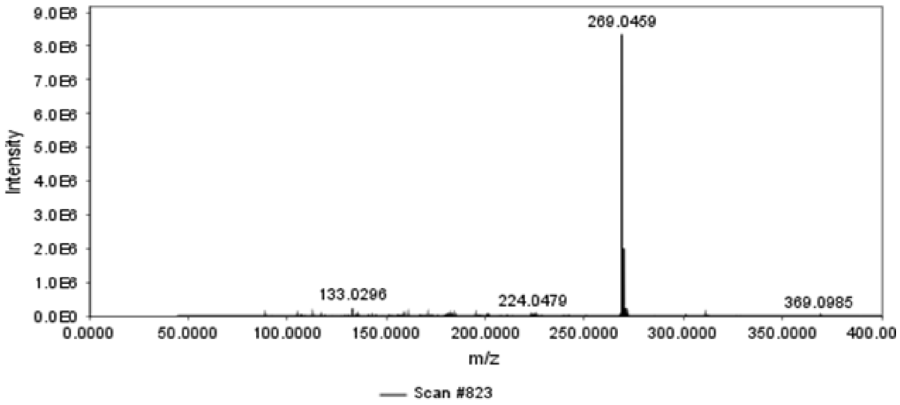

Figure S15. Mass spectrum corresponding to genistein (peak 50, Rt: 14.30 min) in sub-fraction D8.

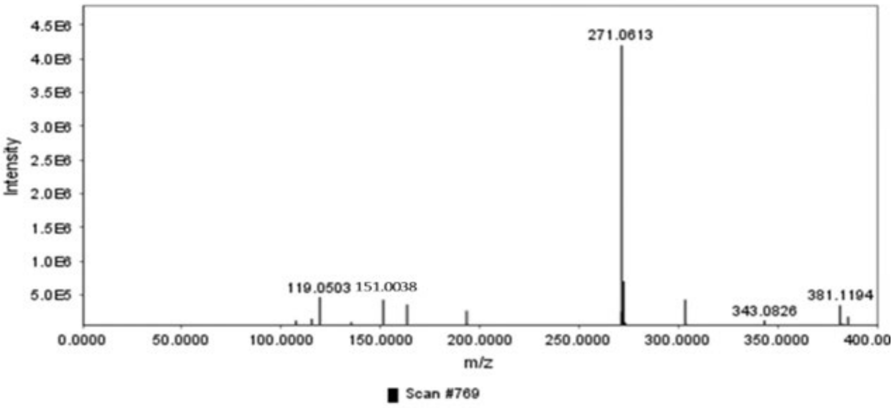

Figure S16. Mass spectrum corresponding to naringenin (peak 47, Rt: 13.30 min) in sub-fraction D8.

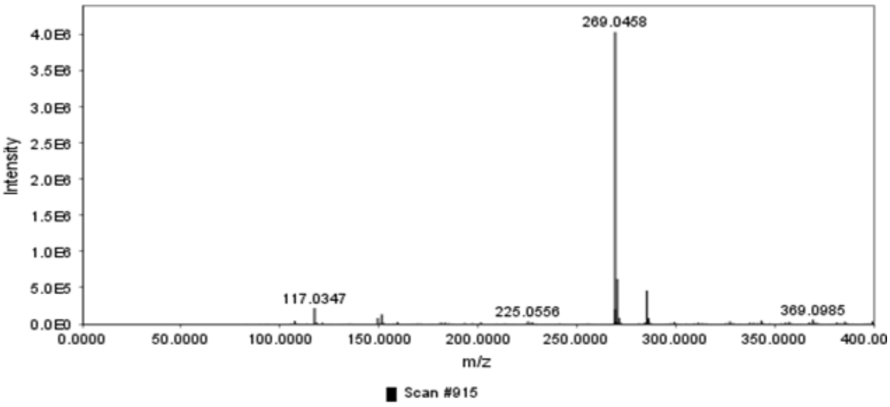

Figure S17. Mass spectrum corresponding to apigenin (peak 54, Rt: 15.80 min) in sub-fraction D8.

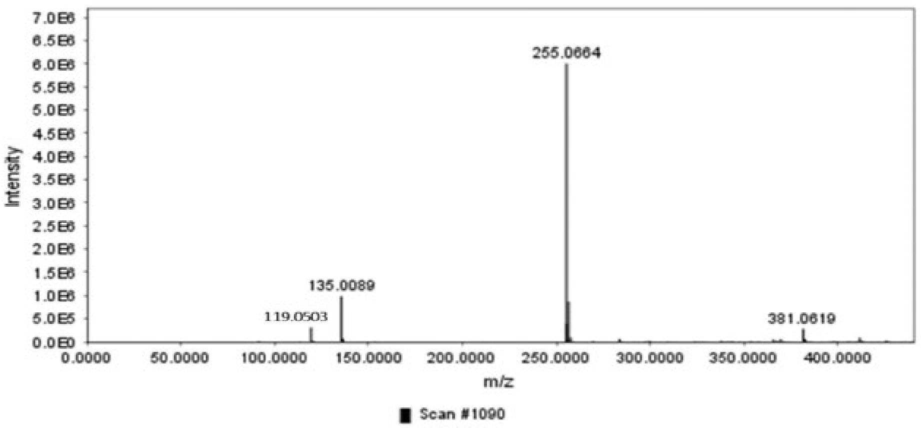

Figure S18. Mass spectrum corresponding to liquiritigenin (peak 62, Rt: 18.80 min) in sub-fraction D8.

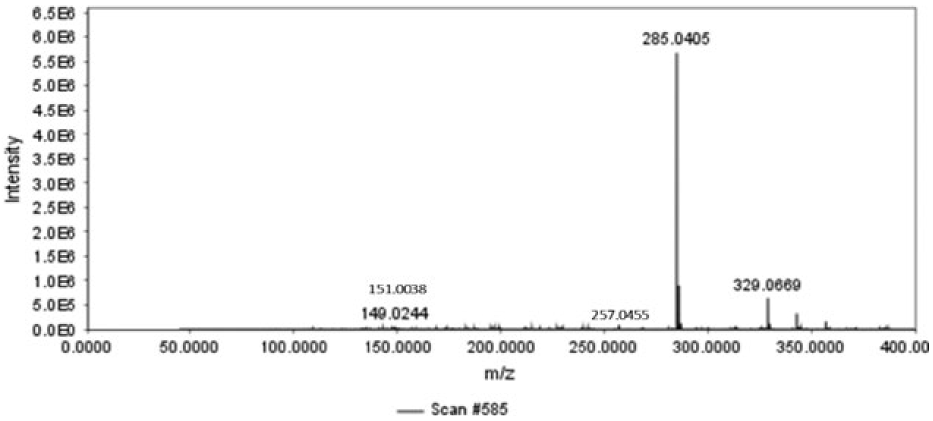

Figure S19. Mass spectrum corresponding to kaempferol (peak 35, Rt: 10.10 min) in sub-fraction D8.

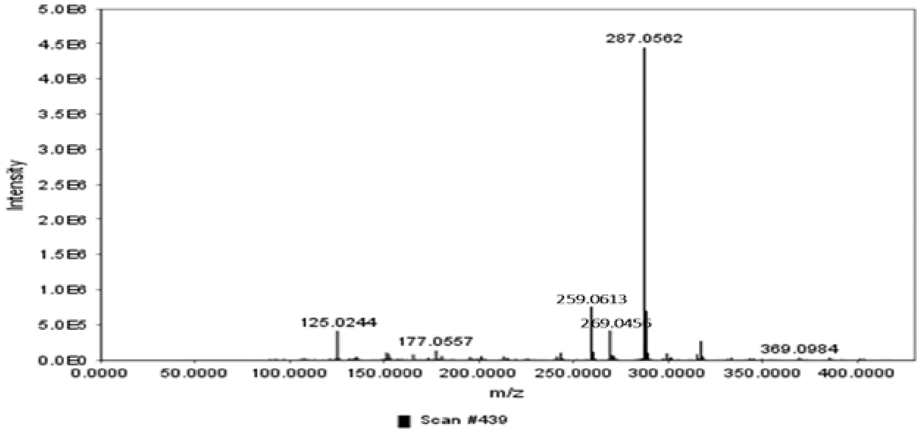

Figure S20. Mass spectrum corresponding to dihydrokaempferol (peak 24, Rt: 7.60 min) in sub-fraction D8.

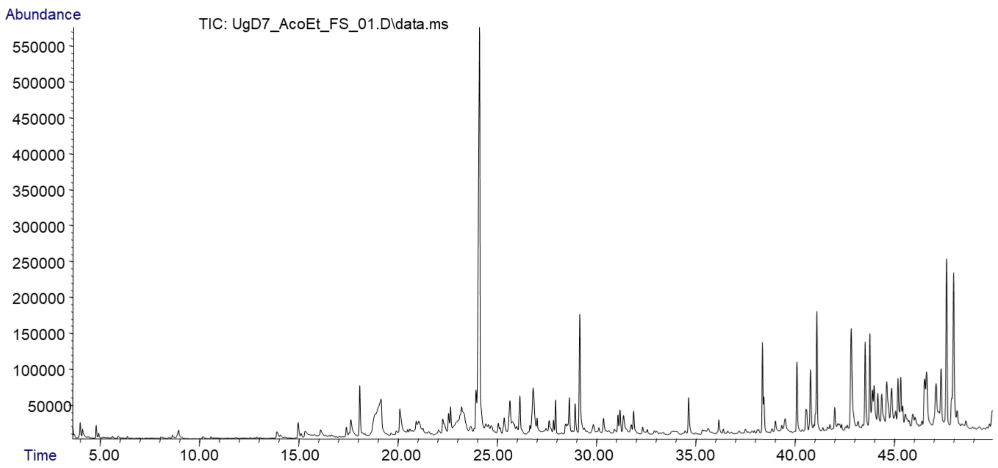

Figure S21. GC-MS chromatogram of sub-fraction D7 of *U. gallii*.

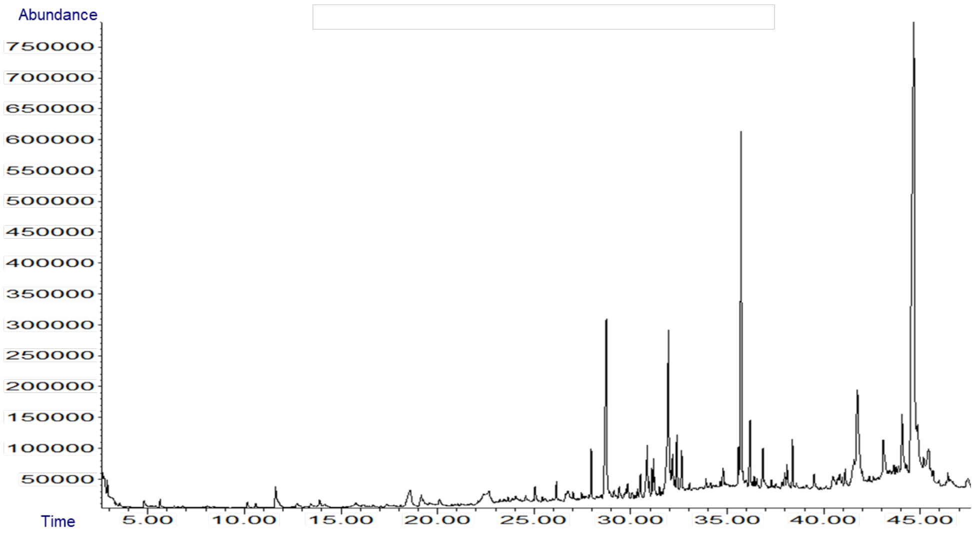

Figure S22. GC-MS chromatogram of sub-fraction D8 of *U. gallii*.

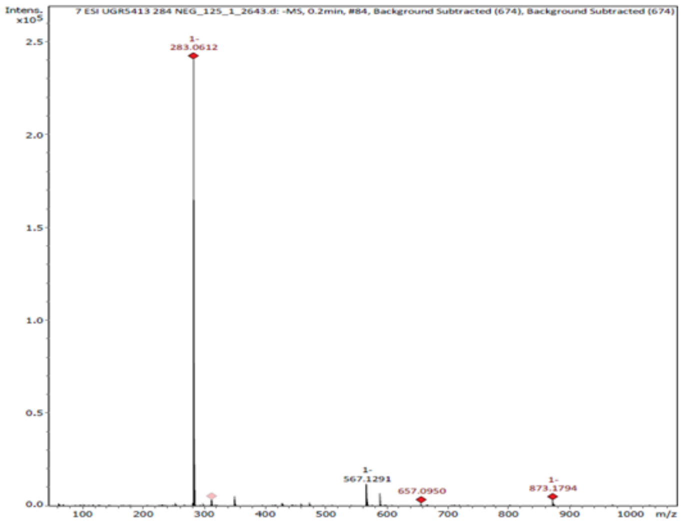

Figure S23. ESI-FIA-TOF spectrum of isoprunetin (**C1**).

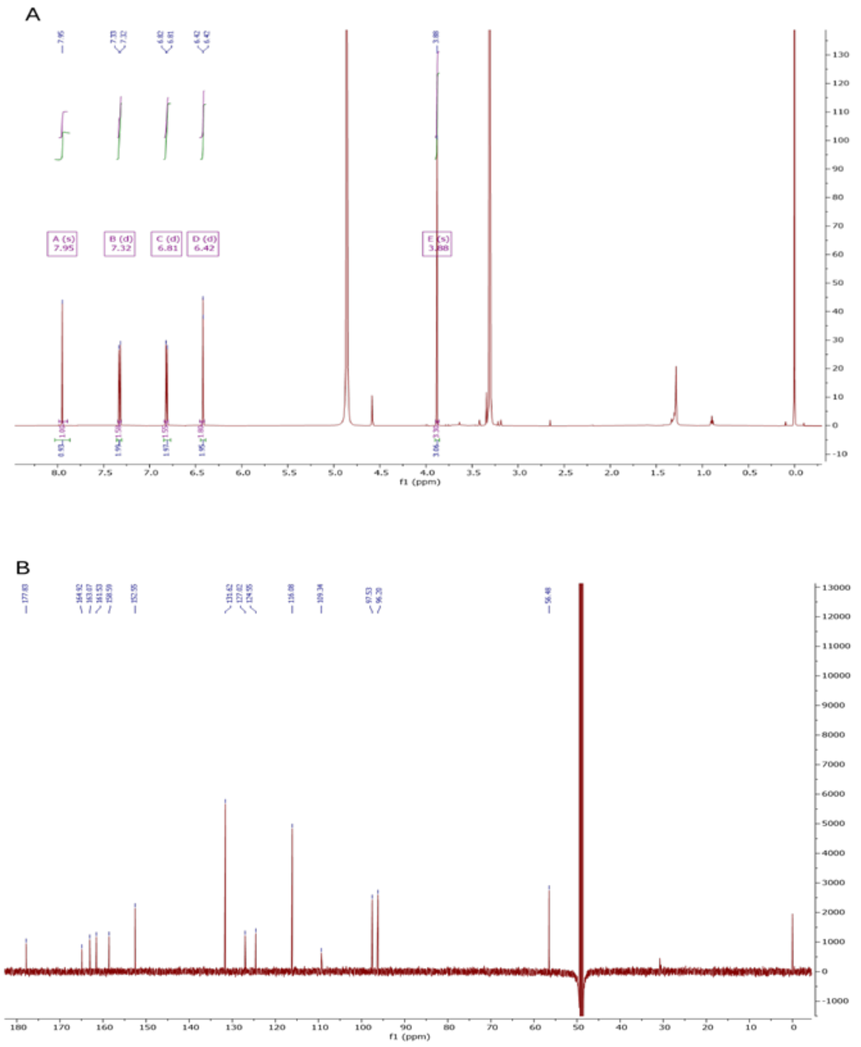

Figure S24. ^{1}H NMR(A) and ^{13}C NMR (B) spectra of **C1** in CD_3OD.

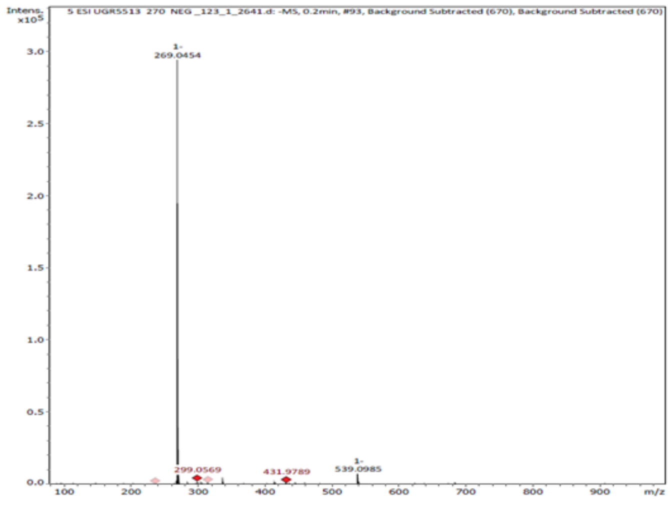

Figure S25. ESI-FIA-TOF spectrum of genistein (**C2**).

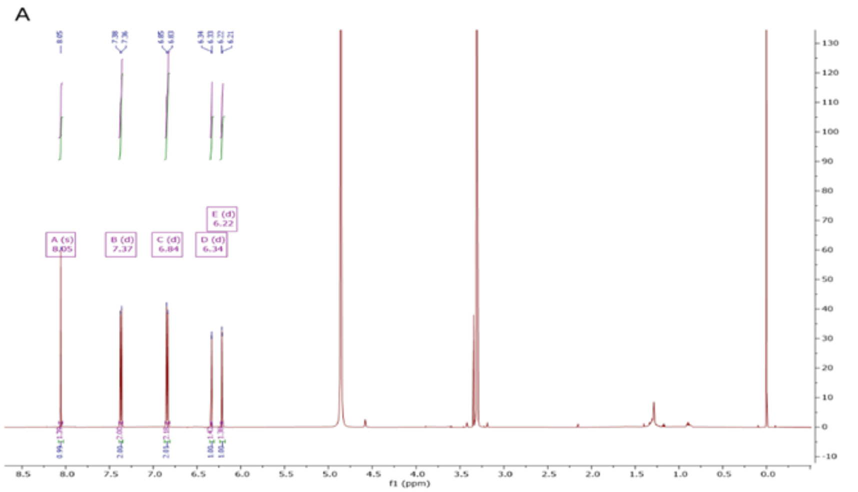

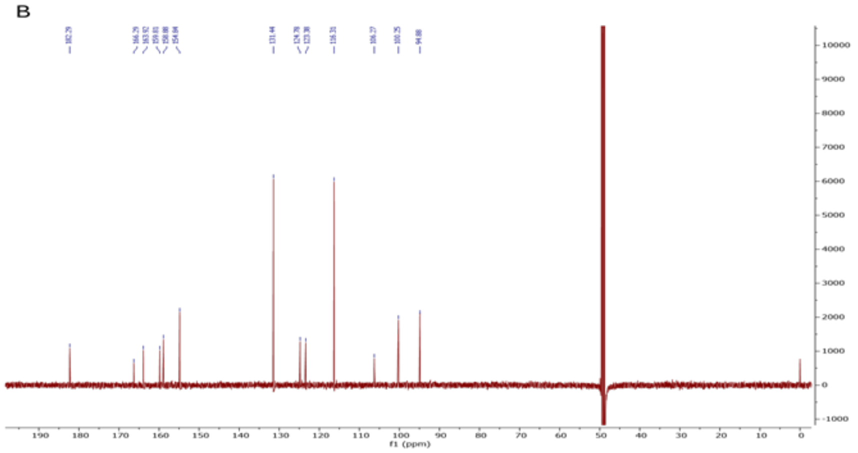

Figure S26. ^{1}H NMR (A) and ^{13}C NMR (B) spectra of **C2** in CD_3OD.

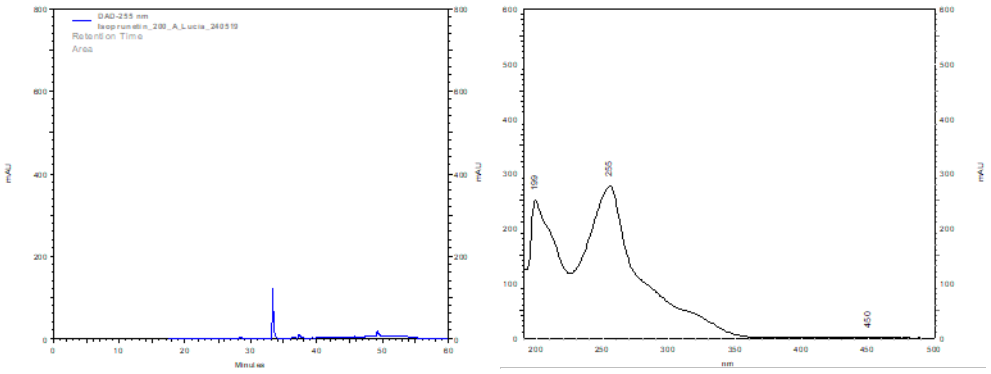

Figure S27. HPLC chromatogram at 255 nm (left) and UV spectrum (right) of isoprunetin (**C1**).

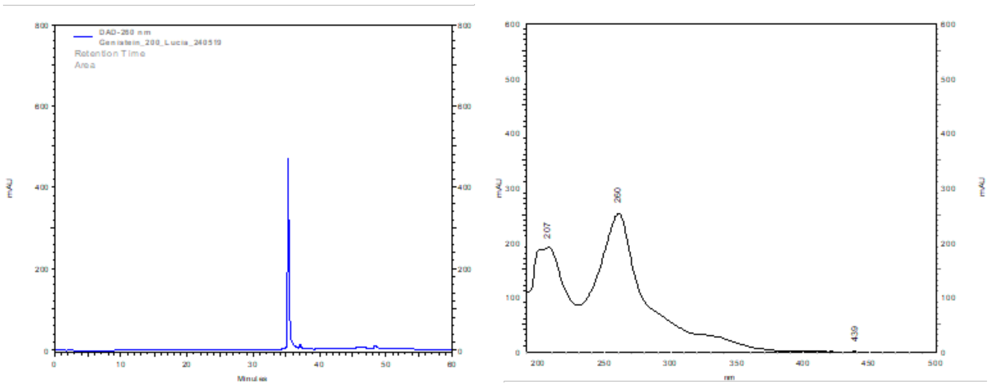

Figure S28. HPLC chromatogram at 260 nm (left) and UV spectrum (right) of genistein (**C2**).

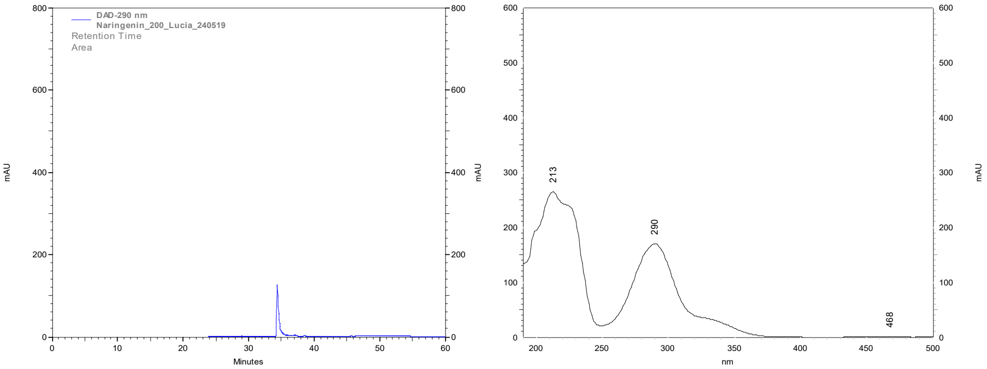

Figure S29. HPLC chromatogram at 290 nm (left) and UV spectrum (right) of naringenin.

References

[1] Bada, L.; Pereira, R.B.; Pereira, D.M.; Lores, M.; Celeiro, M.; Quezada, E.; Uriarte E.; Gil-Longo, J.; Viña D. Phytochemical analysis and antiproliferative activity of *Ulex gallii* Planch. (Fabaceae), a medicinal plant from Galicia (Spain). *Molecules* **2023**, *28*, 351. DOI: 10.3390/molecules28010351.
